# A novel IFNα-induced long noncoding RNA negatively regulates immunosuppression by interrupting H3K27 acetylation in head and neck squamous cell carcinoma

**DOI:** 10.1186/s12943-019-1123-y

**Published:** 2020-01-06

**Authors:** Hailong Ma, Hanyue Chang, Wenyi Yang, Yusheng Lu, Jingzhou Hu, Shufang Jin

**Affiliations:** 1 Department of Oral Maxillofacial-Head and Neck Oncology, Shanghai Ninth People’s Hospital, College of Stomatology, Shanghai Jiao Tong University School of Medicine, No 639, Zhizaoju Rd, Shanghai, 200011 China; 2 National Clinical Research Center for Oral Diseases, Shanghai, 200011 China; 3 Shanghai Key Laboratory of Stomatology & Shanghai Research Institute of Stomatology, Shanghai, 200011 China; 4 Key Laboratory of Oral Medicine, Guangzhou Institute of Oral Disease, Stomatology Hospital of Guangzhou Medical University, Guangzhou, 510140 China

**Keywords:** Interferon alpha, lncMX1–215, Immunosuppression, H3K27 acetylation, HNSCC

## Abstract

**Background:**

Interferon alpha (IFNα) is a well-established regulator of immunosuppression in head and neck squamous cell carcinoma (HNSCC), while the role of long noncoding RNAs (lncRNAs) in immunosuppression remains largely unknown.

**Methods:**

Differentially expressed lncRNAs were screened under IFNα stimulation using lncRNA sequencing. The role and mechanism of lncRNA in immunosuppression were investigated in HNSCC in vitro and in vivo.

**Results:**

We identified a novel IFNα-induced upregulated lncRNA, lncMX1–215, in HNSCC. LncMX1–215 was primarily located in the cell nucleus. Ectopic expression of lncMX1–215 markedly inhibited expression of the IFNα-induced, immunosuppression-related molecules programmed cell death 1 ligand 1 (PD-L1) and galectin-9, and vice versa. Subsequently, histone deacetylase (HDAC) inhibitors promoted the expression of PD-L1 and galectin-9. Binding sites for H3K27 acetylation were found on PD-L1 and galectin-9 promoters. Mechanistically, we found that lncMX1–215 directly interacted with GCN5, a known H3K27 acetylase, to interrupt its binding to H3K27 acetylation. Clinically, negative correlations between lncMX1–215 and PD-L1 and galectin-9 expression were observed. Finally, overexpression of lncMX1–215 suppressed HNSCC proliferation and metastasis capacity in vitro and in vivo.

**Conclusions:**

Our results suggest that lncMX1–215 negatively regulates immunosuppression by interrupting GCN5/H3K27ac binding in HNSCC, thus providing novel insights into immune checkpoint blockade treatment.

## Introduction

Head and neck cancer ranks as the sixth leading malignancy worldwide, with almost 90% of cases classified as head and neck squamous cell carcinoma (HNSCC) [1]. Most HNSCC cases arise from the mucosal lining in the oral cavity, pharynx and larynx and can seriously limit the chewing ability, speech, cosmetology, and even life of patients. Great progress in targeted therapies, such as cetuximab to target epidermal growth factor, has been made in recent decades, especially for advanced HNSCC. Agents targeting anti-programmed cell death-1 (PD-1) and anti-programmed cell death ligand 1 (PD-L1) have revolutionized HNSCC therapy, triggering huge investigations into the formation and regulation of the immunosuppressive tumor microenvironment [2]. Currently, several immune checkpoint molecules have been verified, including cytotoxic T lymphocyte associated antigen (CTLA-4), PD-1, T cell immunoglobulin domain and mucin domain-3 (TIM-3), lymphocyte-activation gene 3 (LAG-3), and T-cell immune receptor with immunoglobulin and ITIM domain (TIGIT) [3]. Galectin-9 on tumor cells is considered the major ligand for TIM-3 and induces T lymphocyte exhaustion or apoptosis and regulation of NK cell function [4, 5]. However, immune checkpoint blockade (ICB) using anti-PD-1/PD-L1 treatment only leads to a modest overall response rate of ~ 15% [6]. Furthermore, severe immune-related adverse events and cardiotoxicity also hinder its curative effect [7, 8]. Therefore, a comprehensive study of the development of immunosuppression is critical for ICB treatment in HNSCC.

Our previous study demonstrated that endogenous interferon alpha (IFNα)-induced PD-L1 and PD-1 expression is a novel immunosuppression mechanism in HNSCC [9]. However, classic signal transducer and activator of transcription 1 (Stat1) signaling only plays a partial role in the immunosuppression mediated by IFNα. Whether there are certain molecules involved in positive or negative regulation of the immunosuppression mediated by IFNα is still unclear. Over the past years, many long noncoding RNAs (lncRNAs) have emerged as critical regulators in various physiological and pathological processes, especially in the immune response [10]; for example, a host-derived, IFN-inducible lncRNA, lnc-Lsm3b, can compete with viral RNAs in the binding of RIG-I monomers and exert feedback to inactivate the innate RIG-I function at the late stage of the innate response [11]. In addition, lncRNA-ACOD1, a lncRNA induced by multiple viruses but not by type I interferon, facilitates viral replication in mouse and human cells [12]. Accumulating lncRNAs involved in interferon response have been identified, such as lncRNA RP11-2B6.2 [13], lncRNA IRF1-AS [14], lncLrrc55-AS [15], and lnczc3h7a [16]. However, the regulation and function of lncRNAs in IFNα-induced immunosuppression remain unknown.

Herein, we identified the novel IFNα-induced lncRNA MX1–215 (*ENST00000486275*), which negatively regulated immunosuppression formation. H3K27 acetylation activated the expression of two immune checkpoint molecules, PD-L1 and galectin-9, on tumor cells. Mechanistically, we found that lncMX1–215 directly interacted with GCN5, a known H3K27 acetylase, to interrupt its binding to H3K27 acetylation. In addition, lncMX1–215 was significantly downregulated in HNSCC tissues and regulated cell proliferation and migration both in vitro and in vivo. Therefore, our results indicate that lncMX1–215 plays a critical role in immunosuppression development and acts as a tumor suppressor molecule in HNSCC, which highlights a novel regulatory mechanism underlying immunosuppression and tumor progression.

## Methods

### RNAscope, fluorescence in situ hybridization (FISH), immunohistochemistry and immunofluorescence

A tissue microarray (TMA) was prepared using 70 HNSCC and 18 normal oral mucosa tissues from patients who had undergone surgery between 2007 and 2008 and who were diagnosed by pathological examination as previously described [17]. This study was approved by the Ethics Committee of the Ninth People’s Hospital, Shanghai Jiao Tong University School of Medicine.

The RNAscope probe targeting lncMX1–215 was designed and synthesized by Advanced Cell Diagnostics company, and detection of lncMX1–215 expression in the TMA was performed using an RNAscope 2.5 High Definition (HD)-RED Assay kit according to the manufacturer’s instructions (Advanced Cell Diagnostics, Newark, CA, USA). Images were acquired and analyzed with CaseViewer software (3D HISTECH Ltd., Budapest, Hungary).

FISH was conducted according to the manufacturer’s instructions (Ribobio Company, Guangzhou, China). Briefly, a lncMX1–215-specific probe was designed, synthesized and incubated with samples overnight at 4 °C. 18S RNA and U6 were used to identify the cytoplasm and nucleus.

Immunohistochemistry (IHC) and immunofluorescence were performed as described in our previous study [18], with primary antibodies against PD-L1, galectin-9, H3K27ac and GCN5 (Cell Signaling Technology, Danvers, MA, USA), and Ki-67 (Proteintech, Rocky Hill, NJ, USA). The staining intensity was assessed as follows: 0 = negative; 1 = weak; 2 = moderate; 3 = strong. The staining score was calculated by multiplying the staining intensity and the percentage of positive cells.

### Cell lines, plasmid, antisense oligonucleotide (ASO) and siRNA transfection

The HNSCC cell lines HN4, HN6 and HN30 were provided by University of Maryland. Cal27, SCC4, SCC25, Detroit 562 and 293 T cells were purchased from Type Culture Collection of Chinese Academy of Sciences (Shanghai, China). SCC7, a mouse-derived HNSCC cell line, was donated by Prof. Liu from Soochow University. All cell lines were verified by STR genotyping. Normal oral primary keratinocytes (NOKs) were cultured from gingival tissues after tooth extraction from healthy patients. SCC4 and SCC25 cells were maintained in Dulbecco’s modified Eagle’s medium (DMEM)/F12, and the other cell lines were maintained in DMEM (Gibco, Grand Island, NY, USA) supplemented with 10% fetal bovine serum, 1% glutamine, and 1% penicillin-streptomycin. Cells were cultured in a standard humidified atmosphere of 5% CO_2_ at 37 °C. SCC7 cells were cultured in RPMI 1640 medium supplemented with 10% FBS and penicillin/streptomycin (Gibco, Grand Island, NY, USA) at 37 °C with 5% CO_2_.

Human lncMX1–215 expression vector and luciferase plasmids were constructed by BioLink (Shanghai, China). A lncMX1–215-specific ASO was designed and synthesized by Ribobio (Guangzhou, China). siRNA targeting Galectin-9 was designed and synthesized by Biotend (Shanghai, China). Cells were transfected with siRNAs or plasmids using Lipofectamine™ 3000 and RNAiMAX (Invitrogen, Carlsbad, CA, USA) according to the manufacturer’s instructions. Treatments were administered 24 h after transfection. The sequences are provided in Additional file 1: Table S1.

### LncRNA sequence and data availability

LncRNA sequencing was performed using the HiseqXTen platform (Sangon Biotech, Shanghai, China). Differentially expressed lncRNAs were screened based on fold-change values, and *P* values were calculated using *t*-tests. The thresholds set for differential gene expression were a fold change ≥2.0 and a *P* value ≤0.05. Our human lncRNA sequencing data have been approved and assigned the GEO accession number GSE138147. You may view the GSE138147 study at: https://www.ncbi.nlm.nih.gov/geo/query/acc.cgi?acc=GSE138147.

### 5′ and 3′ rapid amplification of cDNA ends (RACE) analysis

Briefly, RACE analyses were conducted with 1 μg of total RNA or polyA+ RNA using a 5’RACE adaptor, Bulge-LoopTM miRNA qRT-PCR Starter Kit and miDETECT A TrackTMmiRNA qRT-PCR Starter Kit (Ribobio, Guangzhou, China) according to the manufacturers’ instructions. The primers used for nested PCR are presented in Additional file 1: Table S1.

### Cytoplasmic and nuclear RNA isolation and qRT-PCR analysis

Cytoplasmic and nuclear RNA was isolated using an RNA Purification Kit (Norgen Biotek, Thorold, ON, Canada) according to the manufacturer’s instructions. qRT-PCR was conducted using a StepOnePlus Real-time PCR system (Thermo Fisher, Waltham, MA, USA) following the manufacturer’s instructions (Takara, Dalian, China). The primer sequences are listed in the Additional file 1: Table S1.

### ChIP

The chromatin immunoprecipitation (ChIP) assay was performed according to the protocol of a SimpleChIP Enzymatic Chromatin IP kit (CST, Danvers, MA, USA) as described in our previous study [17]. Briefly, after indicated treatment, the cells were fixed, lysed and sonicated to appropriate fragments. The prepared chromatin was precipitated using specific antibodies overnight. Then, the binding complexes were thoroughly washed, eluted, purified and analyzed by qPCR. The promoter primers for lncMX1–215, PD-L1 and galectin-9 were synthetized by Sangon Biotech (Shanghai, China) and are described in Additional file 1: Table S1.

### Dual-luciferase reporter assay

Dual-luciferase reporter assays were performed as previously described [19]. Briefly, 293 T cells were cotransfected with each promoter-luciferase construct and a pRL-TK Renilla luciferase construct as an internal control (30:1) for 36 h. Whole-cell lysates were extracted, and luciferase activity was detected using a dual luciferase reporter assay system (Beyotime, Shanghai, China) according to the manufacturer’s instructions.

### Western blotting and immunoprecipitation analysis

Western blotting was performed as described in our previous study [20]. IFNα and recombinant human galectin-9 were purchased from PeproTech (Rocky Hill, NJ, USA). SAHA, MS-275 and fludarabine were purchased from Selleck (Houston, TX, USA). The antibodies used in this study were as follows: PD-L1, Caspase-3 (13847) and cleaved Caspase-3 (32042) antibodies were purchased from Abcam (Cambridge, MA, UK). Stat1, Galectin-9, H3K27ac, H3K9ac, GCN5, PARP and DYKDDDDK antibodies were from Cell Signaling Technology (Danvers, MA, USA). GAPDH, β-actin and α-tubulin antibodies (60004–1, Proteintech, Rocky Hill, NJ, USA) were used as an internal controls. Immunoreactive bands were scanned and analyzed using an Odyssey Infrared Imaging System (LI-COR Biosciences, Lincoln, NE, USA).

Immunoprecipitation was achieved by incubation of anti-GCN5 monoclonal antibody with cell lysate at 4 °C overnight with rotation. The reaction mixture was further incubated with Protein G Magnetic beads for 2~4 h. The precipitated proteins were detected by immunoblotting with the corresponding antibodies.

### NK cell lysis assay

NK cell lysis assays were performed as previously described [9]. After being transfected for 24 h, HN4 and Cal27 cells were seeded in 96-well plates at 4~6 × 10^3^ cells per well. The adherent cells were cocultured with NK cells at different effector-to-target (E:T) cell ratios (as indicated) for 4~6 h. Tumor cell lysis was measured with an LDH cytotoxicity assay kit (Dojindo, Kumamoto, Japan).

### Flow cytometry

Flow cytometry was performed according to the manufacture’s protocol [21]. HN4 and Cal27 cells transfected with lncMX1–215 or vector were harvested 48 h after transfection. After incubation with reagents from an Annexin V-FITC / propidium iodide (PI) apoptosis kit (BD Biosciences), cells were analyzed using a BD Beckman cytometer (BD Biosciences, Franklin Lakes, NJ, USA) and FlowJo software. For cell cycle analysis, cells were incubated with reagents from a PI / RNase staining kit (BD Biosciences). The cells were collected and incubated with anti-human PD-L1-PE antibody (BD Biosciences, Franklin Lakes, NJ) at 1:100 for 30 min on ice. Then, the cells were analyzed on a BD Beckman cytometer.

### RNA immunoprecipitation (RIP) analysis

RNA immunoprecipitation (RIP) assays were performed according to the instructions of a Millipore Magna RIP Kit (Millipore, Darmstadt, Germany). HN4 cells were transfected with recombinant DYKDDDDK-GCN5 fusion plasmid for 24 h and harvested. The cell lysates were incubated with RIP buffer containing magnetic beads conjugated with IgG and DYKDDDDK tag antibodies overnight at 4 °C. The samples were then incubated with proteinase K to isolate immunoprecipitated RNA. The isolated RNA was analyzed via qRT-PCR, which was performed using SYBR Green (Takara, Dalian, China) with primers targeting lncMX1–215 truncations (Additional file 1: Table S1).

### RNA pull-down assay

RNA pull-down assays were performed according to the instructions provided in a Pierce™ Magnetic RNA-Protein Pull-Down Kit (Thermo Scientific, Waltham, MA, USA). LncMX1–215 RNA was transcribed in vitro using a DNA template that included the T7 promoter according to the instructions of a Ribo™ RNAmax-T7 Transcription Kit (RiboBio, Guangzhou, China). Then, lncMX1–215 was end-labeled with desthiobiotin using a Pierce RNA 3′ End Desthiobiotinylation Kit (Thermo Scientific, Waltham, MA, USA). Biotinylated lncMX1–215 was captured with magnetic streptavidin-coated beads and then mixed with HN4 cell extract. The protein was eluted from the RNA protein complexes and detected via western blotting using GCN5 antibody.

### CCK8, 5-Ethyny-2′-deoxyuridine (EdU) assay, clone forming and terminal deoxynucleotidyl transferase dUTP nick end labelling (TUNEL) assay

HN4 and Cal27 cells were transfected with ASO or plasmids of lncMX1–215 for 24 h, and then seeded in the plates. Cell proliferation assay was performed using the Cell Counting Kit (CCK8; Dojindo, Kumamoto, Japan). EdU (RiboBio, Guangzhou, China) was operated according to the manufacture’s protocol. Transfected cells were seed into 6-well plate with 500 cells / well for 10 ~ 14 days to assess the clone-forming capacity. TUNEL (Beyotime, Shanghai, China) was conducted following the manufacturer’s protocol. The apoptotic cells were observed using Axio Vert. A1 fluorescence microscope.

### Transwell assay

Cell migration and invasion assays were performed using Transwell assay with uncoated polycarbonate inserts (Millipore, Darmstadt, Germany) for migration or BioCoat™ inserts (BD Biosciences, Franklin Lake, NJ, USA) for invasion. 1 ~ 5 × 10^4^ transfected cells were seed in the upper chamber. After crystal violet staining, the positive cells were counted and analyzed under microscope.

### In vivo analysis

BALB/c nude mice (aged 4 ~ 6 weeks) purchased from the Shanghai Laboratory Animal Center (Shanghai, China) were bred in SPF facilities. 1 × 10^6^ Cal27 cells stably transduced with lentivirus encoding lncMX1–215 were injected into the left or right flanks of mice for tumorigenicity. 1 × 10^6^ cells were injected through lateral tail vein for lung metastasis assay. Tumor volume was regularly measured, and tumor weight were finally measured after the mice were sacrificed. 1 × 10^5^ SCC7 cells after stably transfection were s.c. inoculated in C3H mice, which was purchased from Beijing Vitalriver company (Beijing, China). Moreover, the transfected SCC7 cells (5 × 10^5^) were injected via lateral tail vein for lung metastasis. The human lncMX1–215 sequence had relative high similarity (~ 80%) with that in mice genome according to UCSC database. Whole lung tissues were removed and fixed in Bouin’s fixative diluted 1:5 with neutral-buffered formalin for metastasis nodules observation. The excised tumor and lung were fixed and paraffin-embedded for staining detection. The in vivo studies were approved by the Animal Care and Use Committee of Ninth People’s Hospital, Shanghai Jiao Tong University School of Medicine.

### Statistical analysis

Statistical analyses were performed using SPSS 22.0 software (SPSS Inc., Chicago, IL, USA). GraphPad Prism 6.0 (GraphPad Software, San Diego, CA, USA) was applied to plot the data. Student’s *t*-test and one-way analysis of variance (ANOVA) were used to assess the significance of differences. *P* < 0.05 was considered statistically significance (* ^or #^ *P* < 0.05, and ** ^or ##^ *P* < 0.01). All values are expressed as the means ± standard error.

## Results

### IFNα-induced lncMX1–215 expression is dependent on p-stat1 in HNSCC cells

**Fig. 1:**
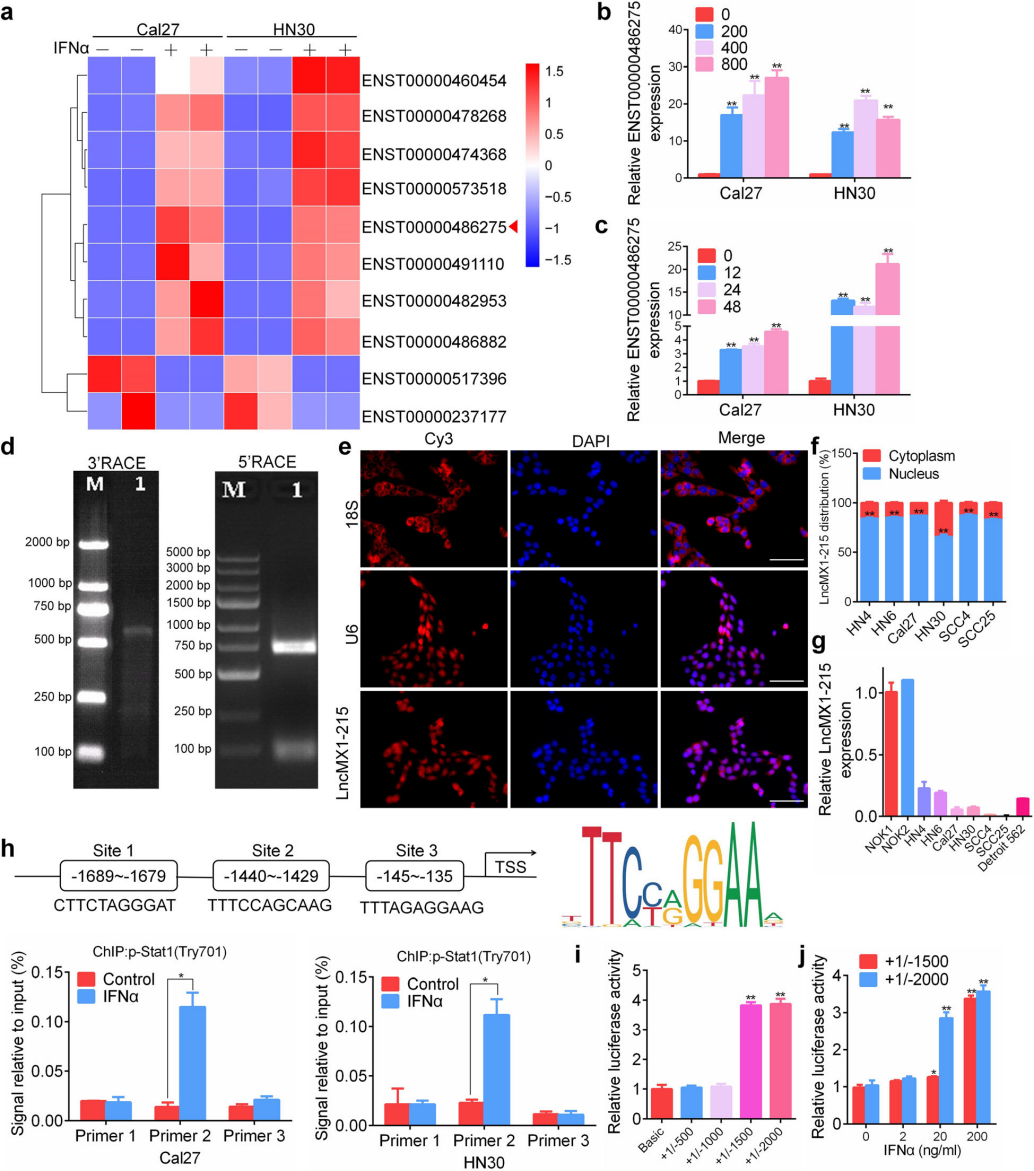
IFNα-induced lncMX1–215 expression is dependent on p-stat1 in HNSCC cells. **a** lncRNA sequencing was performed in Cal27 and HN30 cells after treatment with 200 ng/ml IFNα for 24 h. **b, c** The relative ENST00000486275 expression level under IFNα treatment at the indicated dose and time was detected using qRT-PCR. **d** Agarose gel electrophoresis for 3′ and 5′ RACE analysis of lncMX1–215 was shown. **e** The distribution of lncMX1–215 was analyzed via FISH in HN30 cells. 18S RNA and U6 indicate the cytoplasm and nucleus, respectively. **f** The subcellular distribution of lncMX1–215 was analyzed via PCR in HNSCC cell lines. **g** lncMX1–215 expression was detected in HNSCC cell lines and normal oral keratinocytes. **h** The binding sites and stat1 motif in the promoter region of lncMX1–215 is shown. ChIP assays were performed after treatment with 200 ng/ml IFNα for 24 h. **i** Transcriptional activity was detected using dual-luciferase reporter assays with lncMX1–215 promoter-luciferase truncations in 293 T cells. **j** After transfection of 293 T cells with the indicated promoter truncations for 24 h, the cells were treated with the indicated IFNα stimulation for another 24 h. Then, the transcriptional activity was detected using a dual-luciferase reporter assay. Scale bars, 100 μm; *: *P* < 0.05; **: *P* < 0.01

LncRNA sequencing was conducted to explore the potential lncRNAs involved in the IFNα-related response (GSE138147). After the cells were treated with IFNα, 10 lncRNAs were found to be significantly aberrantly expressed by more than two-fold (Fig. 1a). Secretion of IFNα in the tumor microenvironment was further confirmed with or without chemotherapy stimulation using an ELISA (Additional file 3: Figure. S1). Then, we validated the differential expression of lncRNAs via qRT-PCR in Cal27 and HN30 cells after IFNα treatment (Additional file 3: Figure. S2). ENST00000486275 was the most abundant according to the CT value in the PCR analysis but did not exhibit the greatest difference compared with the control. IFNα could induce the expression of this lncRNA in a dose- and time-dependent manner (Fig. 1b, c). Since ENST00000486275 was the transcript for MX1–215, a retained intron lncRNA, we named it lncMX1–215. 3′ and 5′ RACE was conducted to identify the location and sequence information for lncMX1–215, and we found that it is located on chromosome 21 and has a length of 649 bp (Fig. 1d, Additional file 3: Figure. S3 and S4). The cellular localization of lncRNAs plays a critical role in function investigations. LncMX1–215 was primarily distributed in the nucleus in HNSCC cell lines according to FISH and qPCR assays (Fig. 1e, f, Additional file 3: Figure. S5). Moreover, we observed downregulation of lncMX1–215 in all seven cell lines compared with two primary normal oral keratinocyte lines (Fig. 1g). We also observed a decrease in lncMX1–215 in 3/4 HNSCC patients compared with adjacent normal tissues (Additional file 3: Figure. S6). To explore whether IFNα-induced lncMX1–215 expression depends on stat1 signaling, fludarabine, a stat1 inhibitor, was applied. Fludarabine inhibited IFNα-induced lncMX1–215 expression in Cal27 and HN30 cells (Additional file 3: Figure. S7). Moreover, stat1-specific siRNA could also suppress IFNα-induced lncMX1–215 expression (Additional file 3: Figure. S8). Three binding sites on the promoter region of lncMX1–215 were predicted in JASPAR according to the binding motif of stat1. We found that p-stat1 directly bound to site 2 (− 1440~ − 1429) in lncMX1–215 after IFNα stimulation (Fig. 1h, Additional file 3: Figure. S9), and this binding was decreased after fludarabine treatment (Additional file 3: Figure. S10). Dual-luciferase assays showed that transcriptional activity was notably increased with the + 1/− 1500 truncation and full-length construct after IFNα administration (Fig. 1i), which also indicated that the potential binding sites are located between − 1000 to − 1500. Furthermore, the transcriptional activity was significantly enhanced under 20 and 200 ng/ml IFNα (Fig. 1j). These results demonstrate that IFNα transcriptionally activates lncMX1–215 expression, which is dependent on p-stat1, in HNSCC cells.

### LncMX1–215 negatively regulates IFNα-induced PD-L1 and galectin-9 expression

**Fig. 2:**
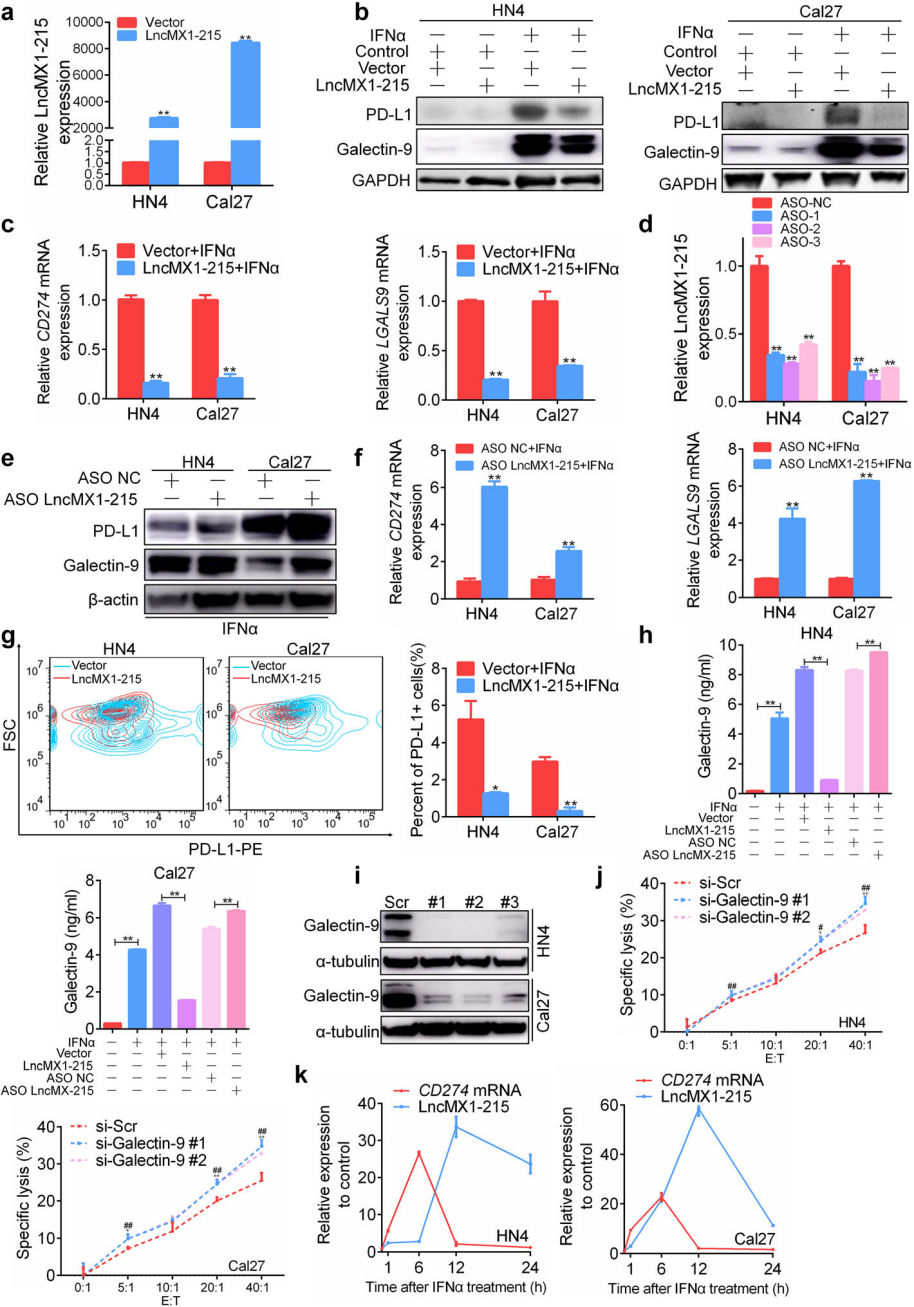
LncMX1–215 negatively regulates IFNα-induced PD-L1 and galectin-9 expression. **a** lncMX1–215 expression was detected in HN4 and Cal27 cells via PCR after transfection with expression plasmids for 24 h. **b, c** The protein and mRNA of PD-L1 and galectin-9 expression levels were detected after transfection with lncMX1–215 for 24 h followed by 200 ng/ml IFNα stimulation for 24 h. **d** The lncMX1–215 knockdown efficiency after transfection with ASO for 24 h was analyzed. **e, f** After ASO transfection for 24 h followed by 200 ng/ml IFNα stimulation for 24 h, PD-L1 and galectin-9 expression levels were detected using western blotting and qRT-PCR. **g** Surface PD-L1 expression was examined using flow cytometry after transfection with lncMX1–215 followed by 200 ng/ml IFNα stimulation for 24 h. **h** The galectin-9 concentration in medium supernatant was measured via ELISA after transfection for 24 h followed by 200 ng/ml IFNα stimulation for 24 h. **i** Galectin-9 knockdown efficiency was detected via western blotting after transfection with siRNA for 48 h. **j** Transfected cells were seeded in a 96-well plate and incubated with NK cells for 4 h at various effector/target (E:T) cell ratios as indicated. The specific lysis rate was measured using an LDH kit. **k** Relative CD274 mRNA and lncMX1–215 expression were detected using qRT-PCR after 200 ng/ml IFNα stimulation for indicated time. *: *P* < 0.05; **: *P* < 0.01

To explore whether lncMX1–215 can affect the immunosuppression mediated by IFNα, ectopic lncMX1–215 expression was confirmed in Cal27 and HN4 cells harboring the highest lncMX1–215 expression (Fig. 2a). We observed that IFNα-induced PD-L1 and galectin-9 expression was significantly inhibited after transfection with lncMX1–215 (Fig. 2b, c). Considering its nuclear localization, an ASO targeting lncMX1–215 was applied to knockdown lncMX1–215 expression (Fig. 2d). Downregulation of lncMX1–215 expression increased PD-L1 and galectin-9 expression under IFNα treatment (Fig. 2e, f). The number of surface PD-L1+ cells also decreased after lncMX1–215 expression under IFNα stimulation according to flow cytometry analysis (Fig. 2g). The galectin-9 concentration in medium supernatant was significantly decreased after overexpression of lncMX1–215 but increased after transfection with ASO under IFNα treatment (Fig. 2h). To further validate the immunosuppression function of galectin-9, its expression was silenced using siRNA targeting galectin-9 (Fig. 2i). HN4 and Cal27 cells were more susceptible to NK cell-mediated immune cell lysis upon galectin-9 silencing (Fig. 2j). Furthermore, recombinant human galectin-9 protected tumor cells from NK cell-mediated lysis (Additional file 3: Figure. S11). Interestingly, we observed that the upregulation of *CD274* mRNA was ahead of lncMX1–215 in HN4 and Cal27 cells (Fig. 2k), which suggested that lncMX1–215 might be a novel feedback loop to balance the immunosupression after IFNα treatment. These results indicate that lncMX1–215 negatively regulates IFNα-induced PD-L1 and galectin-9 expression, which plays a critical role in the immunosuppression network.

### Gain of H3K27 acetylation activates PD-L1 and galectin-9 expression

**Fig. 3:**
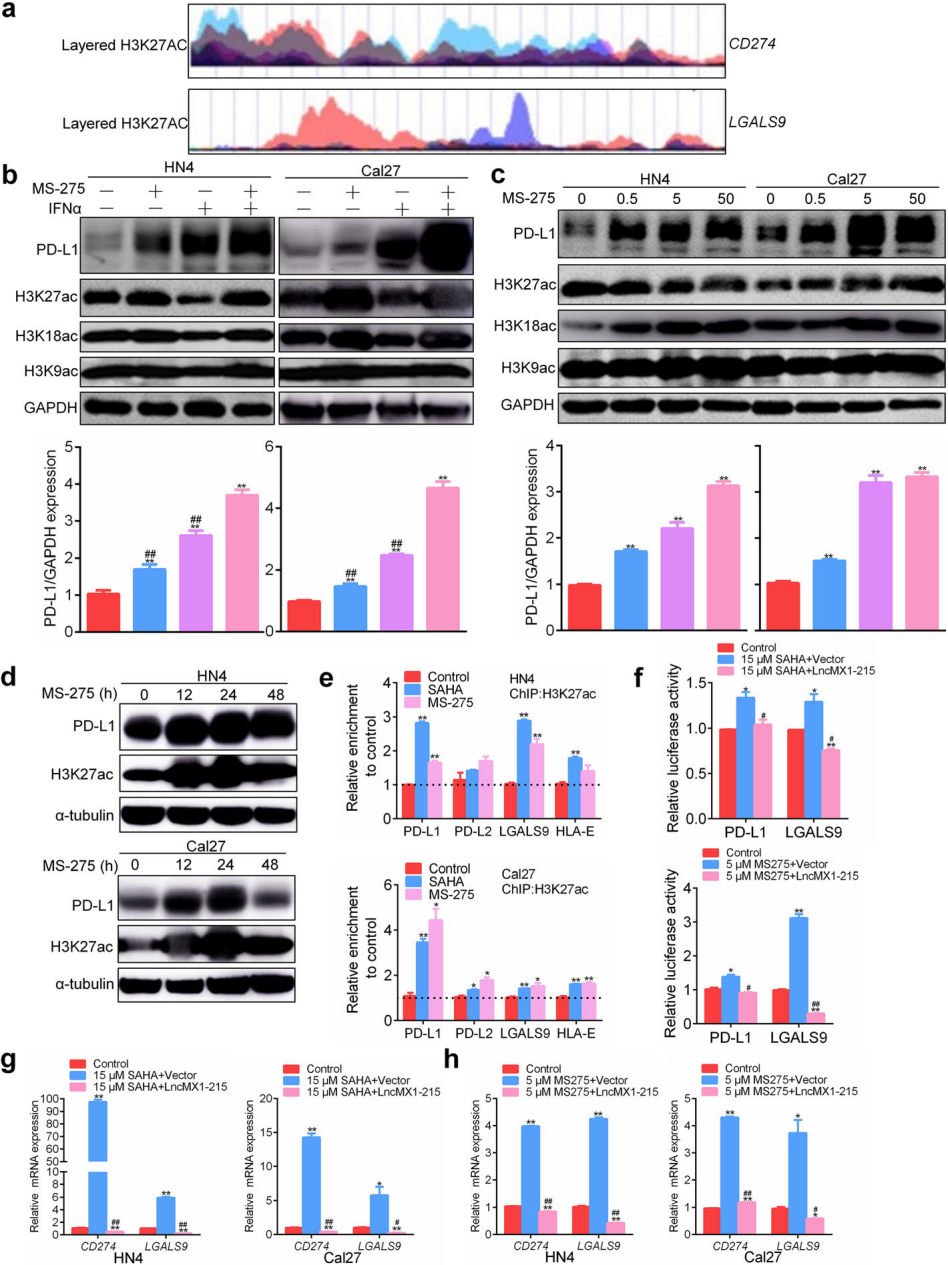
Gain of H3K27 acetylation activates PD-L1 and galectin-9 expression. **a** Layered H3K27 acetylation enrichment in the upstream region of PD-L1 and LGALS9, as shown in the UCSC database. **b** PD-L1 and acetylation of histone 3 were detected and quantified after treatment with 200 ng/ml IFNα or 5 μM MS-275 for 24 h. ^#^ indicated the deference between combined group and each alone. **c** PD-L1, H3K27ac, H3K18ac and H3K9ac were detected and quantified after the indicated MS-275 treatment for 24 h. **d** PD-L1 and H3K27ac were detected and quantified after 5 μM MS-275 treatment for the indicated time. **e** After treatment of HN4 and Cal27 cells with 15 μM SAHA or 5 μM MS-275 for 24 h, ChIP assays were performed using anti-H3K27ac antibody. **f** The promoter activity of PD-L1 and LGALS9 was measured after transfection with lncMX1–215 for 24 h and then SAHA or MS-275 treatment for 24 h in 293 T cells. **g, h** The relative mRNA of *CD274* and *LGALS9* were detected after transfection with lncMX1–215 and then 15 μM SAHA or 5 μM MS-275 treatment for 24 h in HN4 and Cal27 cells. *: *P* < 0.05; **: *P* < 0.01

To explore the possible mechanisms underlying transcriptional regulation of PD-L1 and galectin-9, the UCSC database was searched to find layered H3K27 acetylation enrichment in the upstream region of PD-L1 and LGALS9, which encodes the gelectin-9 protein (Fig. 3a). Two histone deacetylase (HDAC) inhibitors, MS-275and SAHA, were applied to promote histone acetylation. A synergistic effect of the HDAC inhibitors and IFNα on PD-L1 expression was observed in HN4 and Cal27 cells (Fig. 3b, Additional file 3: Figure. S12a). Elevation of H3K27ac was most significant among H3K27ac, H3K18ac and H3K9ac. MS-275 promoted the expression of PD-L1 and H3K27ac in dose- and time-dependent manners (Fig. 3c, d). SAHA promoted PD-L1 expression in dose- and time-dependent manners in Cal27 cells, while slightly decreased at 15 μM and 24 h in HN4 cells (Additional file 3: Figure. S12b, c). Furthermore, the HDAC inhibitors also notably promoted the expression of galectin-9 in a dose-dependent manner (Additional file 3: Figure. S13). According to ChIP assays, binding of H3K27ac and the promoter of four recognized immune checkpoint molecules (PD-L1, PD-L2, LGALS6 and HLA-E) increased under HDAC inhibitor treatment (Fig. 3e). The binding to PD-L1 and LGALS9 was the most obvious. Finally, the transcriptional activity of PD-L1 and LGALS9 was found to increase after HDAC inhibitor treatment, and this increase was reversed after transfection with lncMX1–215 (Fig. 3f, Additional file 3: Figure. S14). Moreover, ectopic expression of lncMX1–215 could significantly reverse the upregulation of CD274 and LGALS9 mRNA mediated by HDAC inhibitors treatment in HN4 and Cal27 cells (Fig. 3g, h). These results indicate that H3K27 acetylation can mediate gene transcription of PD-L1 and LGALS9, while lncMX-215 may disturb this process.

### LncMX1–215 directly interacts with GCN5

**Fig. 4:**
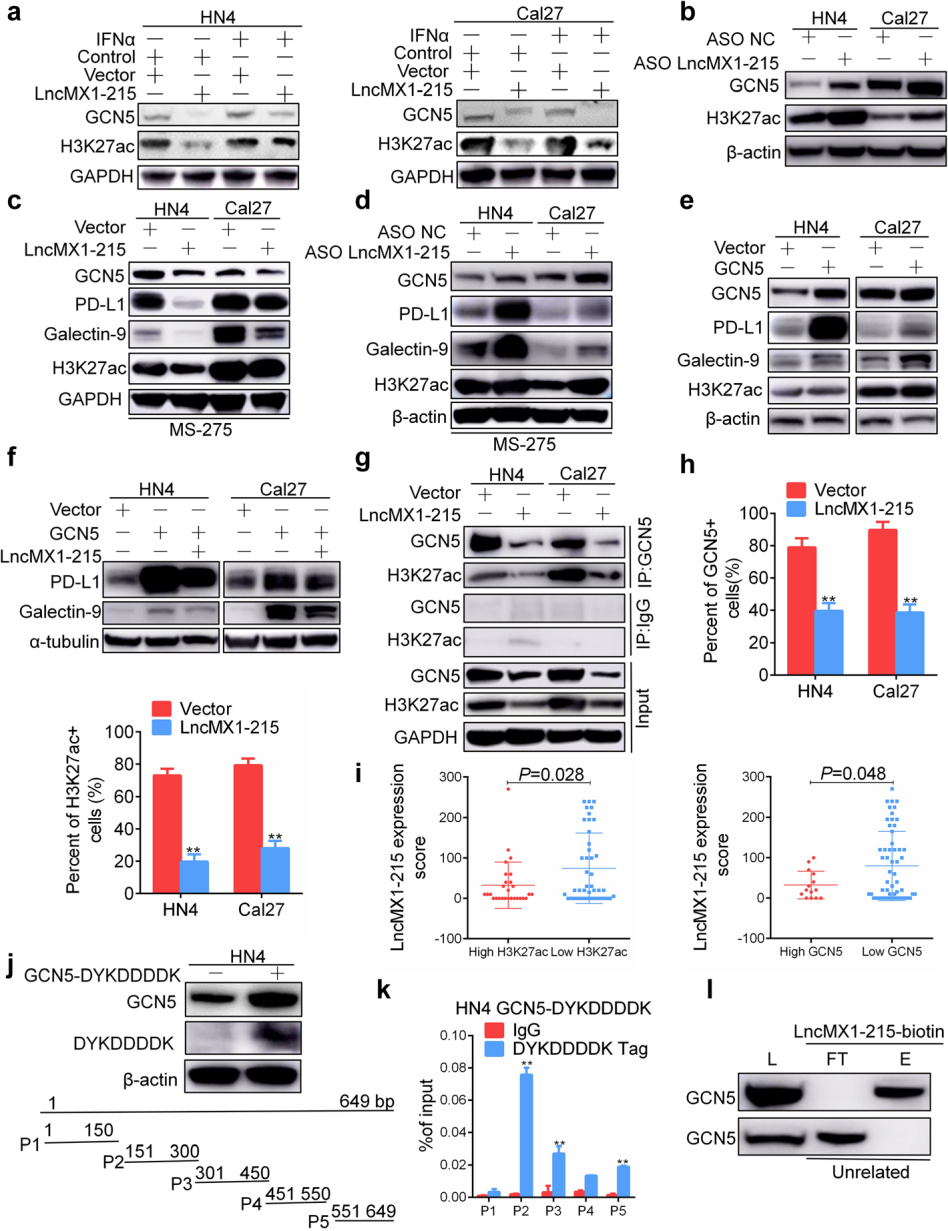
LncMX1–215 directly interacts with GCN5. **a** GCN5 and H3K27ac were detected after transfection with lncMX1–215 for 24 h and treatment with 200 ng/ml IFNα for 24 h. **b** GCN5 and H3K27ac were detected after transfection with ASO for 48 h. **c, d** GCN5, PD-L1, galectin-9 and H3K27ac were examined after transfection with lncMX1–215 or ASO for 24 h followed by treatment with 5 μM MS-275 for 24 h. **e** GCN5, PD-L1, galectin-9 and H3K27ac were detected after ectopic expression of GCN5 for 48 h in HN4 and Cal27 cells. **f** PD-L1 and galectin-9 were detected after cotransfection with GCN5 and lncMX1–215 for 48 h. **g** After transfection with lncMX1–215 for 48 h, cell lysates were precipitated with anti-GCN5 or IgG antibody. **h** GCN5 and H3K27ac expression was analyzed and quantified using immunofluorescence. **i** The correlation between H3K27ac and GCN5 and lncMX1–215 expression was analyzed in HNSCC tissue microarray. **j** Overexpression of GCN5-DYKDDDDK fusion plasmid in HN4 cells was verified by western blotting. P1 to P5 were truncations for full length of lncMX1–215 amplified by specific primers. **k** After transfection of HN4 cells with GCN5-DYKDDDDK fusion plasmid for 48 h, RNA immunoprecipitation was used to detect binding of GCN5 and lncMX1–215. The binding fragments were detected using PCR with primers P1 to P5. **l** Cell lysates were incubated with biotinylated lncMX1–215 or an unrelated probe, and the eluent and flow-through were analyzed via western blotting with anti-GCN5 antibody. L: lysate load; FT: flow-through; E: eluent. *: *P* < 0.05; **: *P* < 0.01

GCN5, a well-known histone acetyltransferase, is primarily responsible for acetylating H3K27. We speculated that lncMX1–215 might interact with GCN5 to impede the acetylation process. We first found that lncMX1–215 significantly inhibited GCN5 and H3K27ac expression independent of IFNα treatment (Fig. 4a), while knockdown of lncMX1–215 expression using an ASO enhanced GCN5 and H3K27ac expression (Fig. 4b). Moreover, GCN5, H3K27ac, PD-L1 and galectin-9 were notably repressed after ectopic expression of lncMX1–215 under MS-275 treatment (Fig. 4c). Conversely, knockdown of lncMX1–215 expression promoted acetylation and PD-L1 and galectin-9 expression (Fig. 4d). To validate the acetylation activity of GCN5, ectopic expression of GCN5 was induced to increase H3K27ac, PD-L1 and galectin-9 expression (Fig. 4e). Overexpression of lncMX1–215 reversed the PD-L1 and galectin-9 expression mediated by GCN5 ectopic expression (Fig. 4f). Therefore, we hypothesized that lncMX1–215 might disturb the binding between GCN5 and H3K27ac. Using immunoprecipitation assays, we confirmed that the presence of lncMX1–215 hindered direct binding of GCN5 to H3K27ac (Fig. 4g), which disturbed acetylation and chromatin opening. After ectopic expression of GCN5 in HN4 and Cal27 cells, ChIP assays revealed that lnMX1–215 could inhibit binding to PD-L1 promoter region of GCN5 (Additional file 3: Figure. S15). These results were also supported by the decrease in the number of GCN5+ and H3K27ac + tumor cells observed via immunofluorescence after ectopic expression of lncMX1–215 (Fig. 4h, Additional file 3: Figure. S16). Moreover, we observed that high expression of H3K27ac and GCN5 had relative low expression of lncMX1–215 in HNSCC patients and vice versa (Fig. 4i, Additional file 3: Fig. S17). The expression of lncMX1–215 was assessed using RNAscope technique. High expression with H3K27ac and GCN5 correlated with advanced TNM stage in HNSCC patients (Additional file 2: Table S2, *P* < 0.05). Together, these results indicate that it is very likely that lncMX1–215 directly binds to GCN5. Thus, RIP was performed to detect whether GCN5 can bind to an lncMX1–215 fragment. Due to the lack of an anti-GCN5 antibody for RIP, we constructed the fusion vector GCN5-DYKDDDDK to utilize the DYKDDDDK tag for application in RIP (Fig. 4j). Based on the RIP assay results, GCN5 directly binds to lncMX1–215 at the P2-P5 fragment (Fig. 4k, Additional file 3: Figure. S18). Biotinylated lncMX1–215 was constructed for RNA pull-down assays (Additional file 3: Figure. S19). There was significant GCN5 enrichment in elution solutions with the lncMX1–215-biotin probe but not in unrelated controls (Fig. 4l). These results indicated that lncMX1–215 can directly bind to GCN5 to hinder the binding of GCN5 and H3K27ac, thereby inhibiting transcription of PD-L1 and galectin-9.

### A negative correlation between lncMX1–215 and PD-L1 and galectin-9 expression was observed in HNSCC tissues

**Fig. 5:**
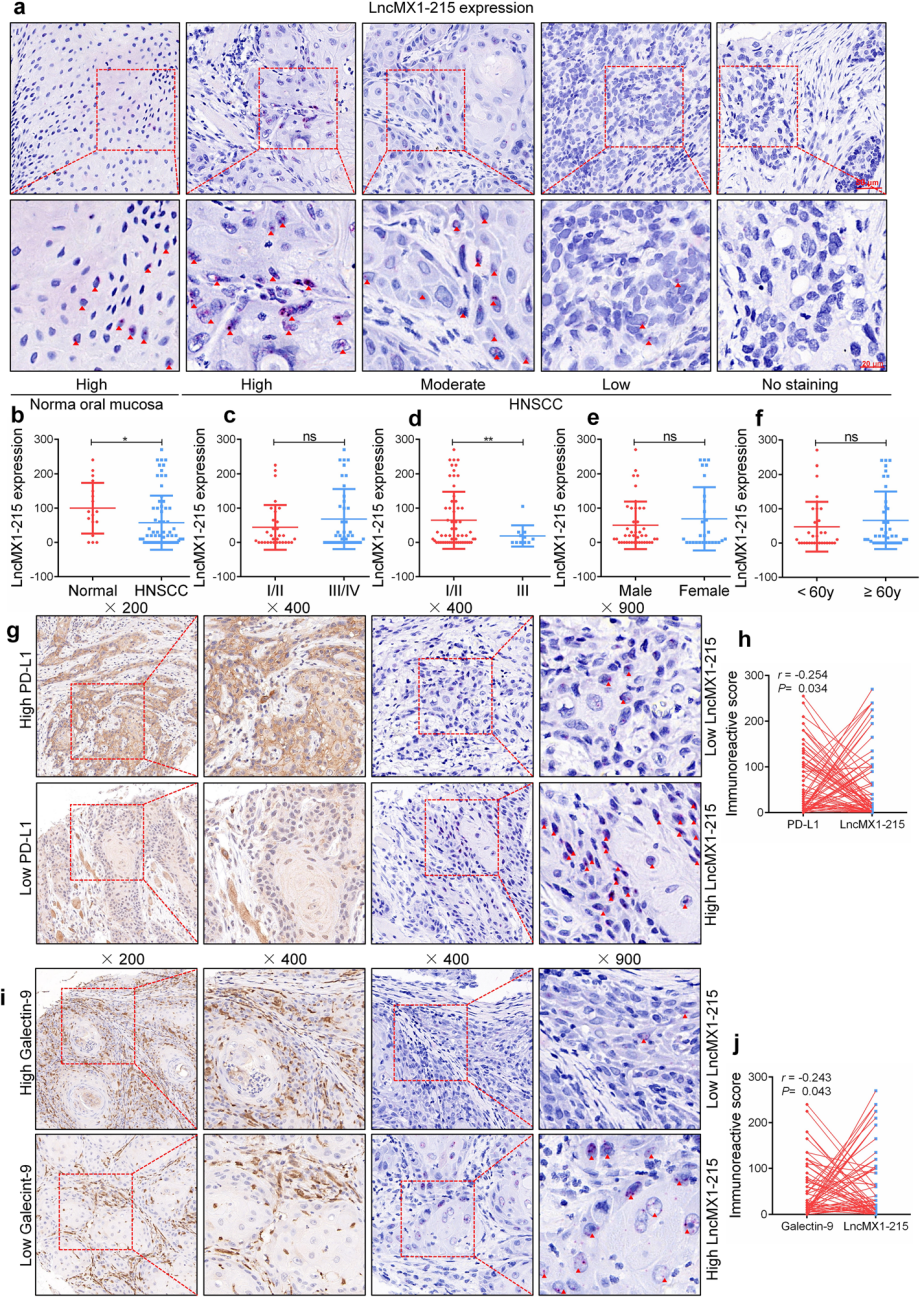
A negative correlation between lncMX1–215 and PD-L1 and galectin-9 expression was observed in HNSCC tissues. **a** Representative images of lncMX1–215 expression in normal and HNSCC tissues shown using the RNAscope technique. **b** The expression scores of lncMX1–215 in normal and HNSCC tissues were compared. **c-f,** Correlations between lncMX1–215 expression and TNM stage, pathological grade, gender and age were analyzed. **g** Representative images of PD-L1 and lncMX1–215 expression were shown. **h** The correlation between PD-L1 and lncMX1–215 expression was analyzed in an HNSCC TMA. **i** Representative images of galectin-9 and lncMX1–215 expression were shown. **j** The correlation between galectin-9 and lncMX1–215 expression was analyzed in an HNSCC TMA. The red triangle indicates an lncMX1–215 molecule. *: *P* < 0.05; **: *P* < 0.01

To explore the correlation between lncMX1–215 and immunosuppression-related molecules, a specific probe for lncMX1–215 was designed and synthesized using RNAscope technology. In Fig. 5a, red dots or dot clusters in the cell nucleus indicate the presence of lncMX1–215. We observed that the expression levels of lncMX1–215 were significantly higher in normal mucosa tissues than in HNSCC tissues (Fig. 5b). Correlations between lncMX1–215 expression and clinical characteristics were also analyzed. LncMX1–215 expression was lower in poor pathological grade tissues than in well/moderate grade tissues (*P* < 0.01, Fig. 5d). However, there was no association between lncMX1–215 expression and TNM stage (Fig. 5c), gender (Fig. 5e) or age (Fig. 5f).

A TMA was used to analyze the correlations between lncMX1–215 expression and that of PD-L1 and galectin-9 in HNSCC tissues. A negative correlation between lncMX1–215 and PD-L1 expression was found (r = − 0.254, *P* = 0.034, Fig. 5g, h). Moreover, a negative correlation between lncMX1–215 and galectin-9 expression was observed in HNSCC tissues (r = − 0.243, *P* = 0.043, Fig. 5i, j). These results demonstrate that lncMX1–215 expression is negatively correlated with immunosuppression in HNSCC clinical samples.

### LncMX1–215 inhibits tumor proliferation capacity in vitro and in vivo

**Fig. 6:**
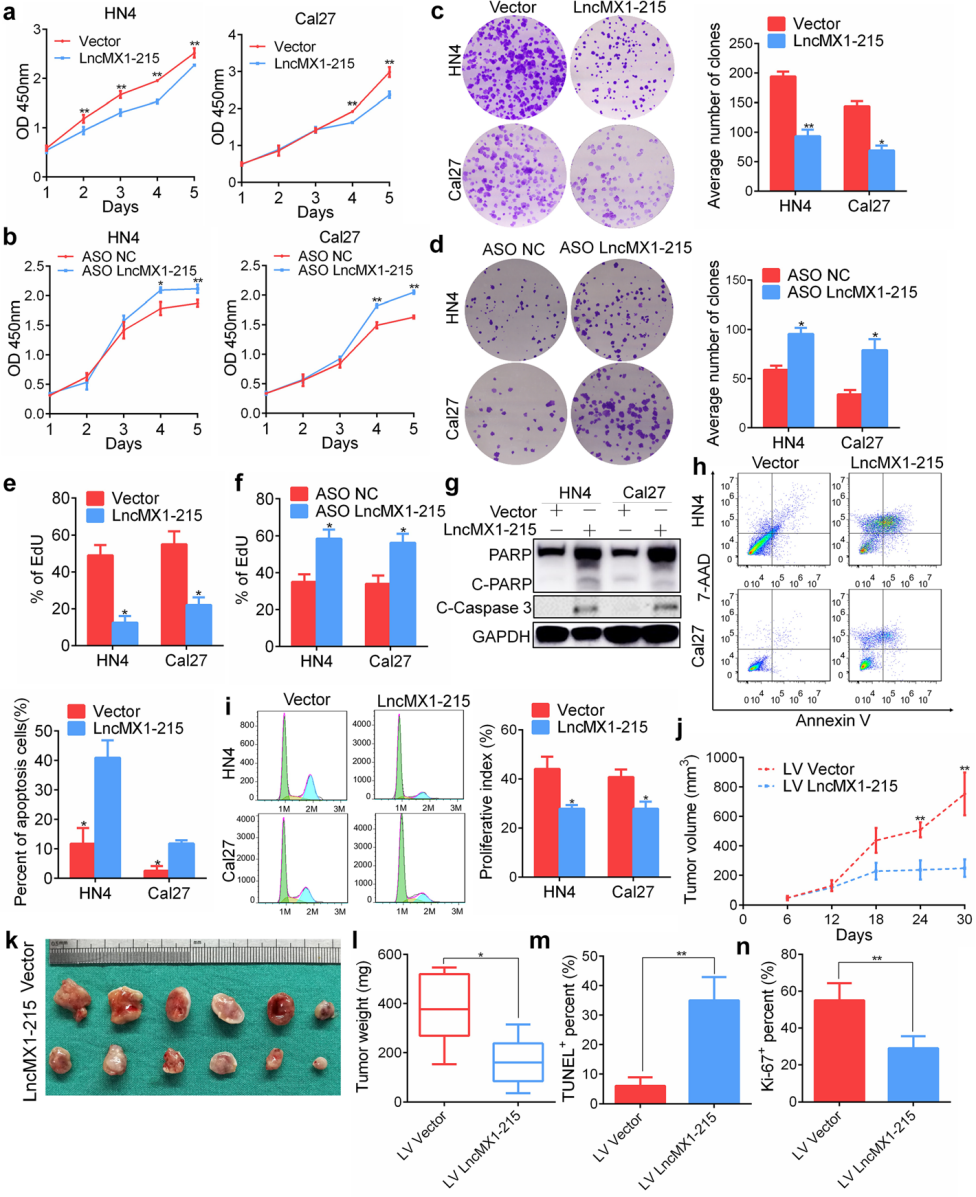
LncMX1–215 inhibits tumor proliferation capacity in vitro and in vivo*.* **a, b** The viability of HN4 and Cal27 cells after transfection with lncMX1–215 or ASO was determined using CCK8 assays. **c, d** Colony formation assays were performed with HN4 and Cal27 cells after transfection with lncMX1–215 or ASO. **e, f** EdU assays were performed after transfection with lncMX1–215 or ASO. **g** PARP and cleaved caspase 3 were detected via western blotting after transfection with lncMX1–215 for 48 h. **h** Cell apoptosis was analyzed via flow cytometry using an Annexin V/7-AAD kit after transfection for 48 h. **i** Cell cycle was analyzed using flow cytometry after transfection for 48 h. **j** Tumor growth curves for mice injected with cells treated with lncMX1–215 lentivirus or vector were analyzed. **k** Tumors derived from the xenograft model were resected and are shown for each group. **l** The weight of tumors resected from mice in the ectopic expression and vector groups was measured and analyzed. **m** The percentage of TUNEL-positive cells in each group was compared. **n** The relative Ki-67 staining score in each group was analyzed. *: *P* < 0.05; **: *P* < 0.01

To explore the biological functions of lncMX1–215, ectopic expression and knockdown models using the ASO were applied. Overexpression of lncMX1–215 significantly inhibited proliferation and colony formation in both HN4 and Cal27 cell lines compared with the scrambled control (Fig. 6a, c). Conversely, knockdown of lncMX1–215 expression enhanced the proliferation and clone formation ability (Fig. 6b, d). In addition, using an EdU assay, inhibition of cell proliferation activity was observed in cells with overexpression of lncMX1–215, and vice versa (Fig. 6e, f, Additional file 3: Figure. S20). Furthermore, the levels of two apoptosis indicators, cleaved-caspase 3 and PARP, increased after transfection with lncMX1–215 (Fig. 6g). The percentage of apoptotic cells was also elevated in lncMX1–215 overexpression cells (Fig. 6h). Based on cell cycle analysis, we observed a decrease in the proliferative index in lncMX1–215 overexpression cells (Fig. 6i).

To validate the anticancer functions of lncMX1–215 in vitro, a xenograft model was constructed. Tumor volume and weight were notably decreased in lncMX1–215 overexpression mice (Fig. 6j, k, l, Additional file 3: Figure. S21). TUNEL assays showed that the number of apoptotic cells in the lncMX1–215 overexpression group was increased (Fig. 6m, Additional file 3: Figure. S22). In addition, Ki67 staining was performed to verify the results (Fig. 6n, Additional file 3: Figure. S23). Moreover, the non-immunosuppressed C3H mice was used to confirm the inhibition of tumorigenesis of lncMX1–215 in SCC7-bearing mice (Additional file 3: Figure. S24a). These findings indicate that lncMX1–215 acts as a tumor suppressor lncRNA in HNSCC.

### Overexpression of lncMX1–215 suppresses HNSCC metastasis

**Fig. 7:**
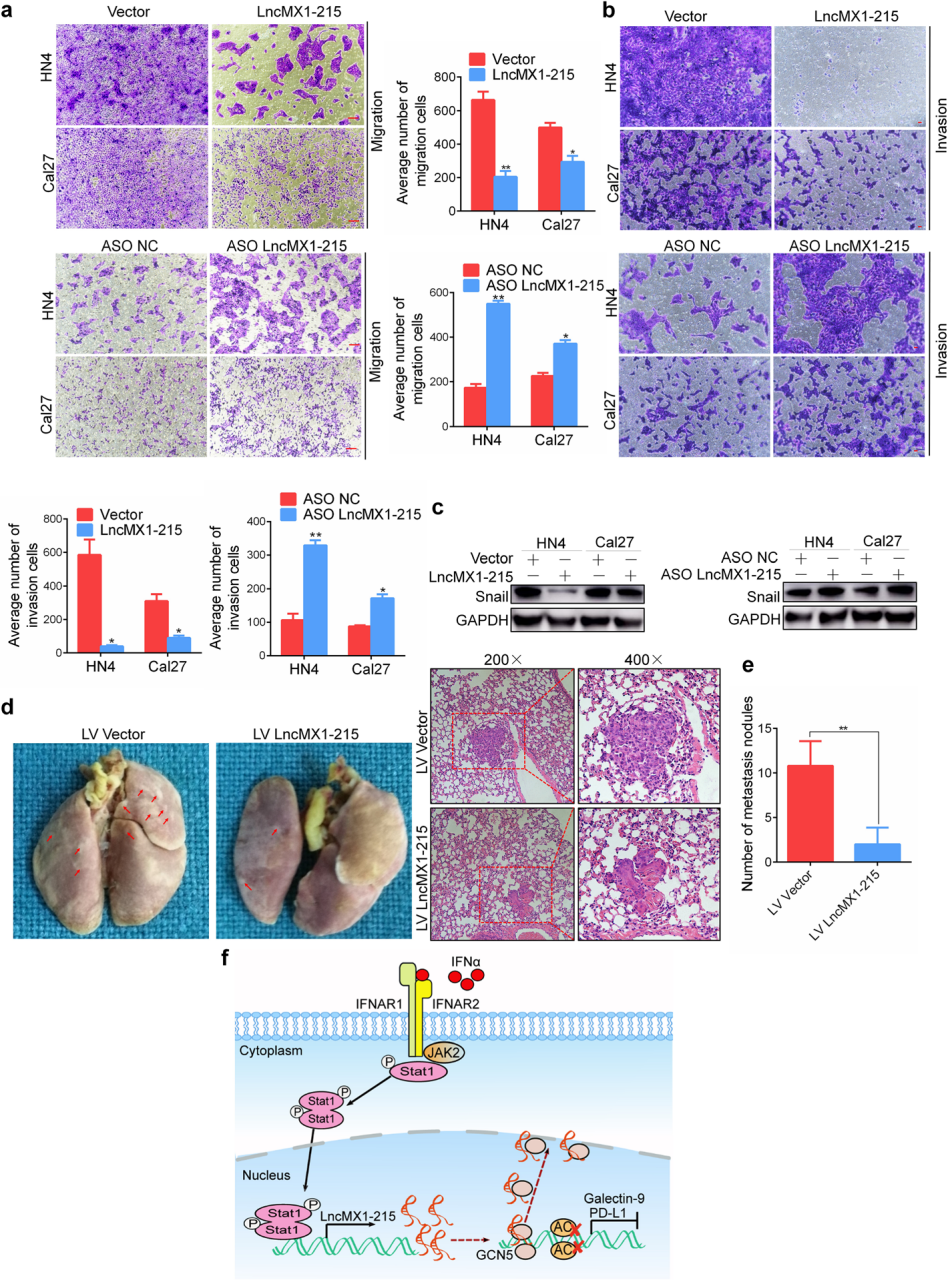
Overexpression of lncMX1–215 suppresses HNSCC metastasis. **a, b** Migration and invasion assays were performed with transfected cells using Transwell inserts (bar = 100 μm, 20 μm, respectively). **c** snail was detected in HN4 and Cal27 cells after transfection with lncMX1–215 or ASO for 48 h. **d** Representative images of lungs from mice in each experimental group. The arrows indicate individual metastatic nodules. Metastatic tumors in lung tissues were identified via hematoxylin and eosin staining. **e** The number of metastatic nodules in each group was analyzed. **f** Schematic diagram showing that IFNα-induced lncMX1–215 can negatively regulate PD-L1 and galectin-9 expression by interrupting GCN5/H3K27ac binding in HNSCC. *: *P* < 0.05; **: *P* < 0.01

Cell migration and invasion capacities were detected using a Transwell insert without or with Matrigel, respectively. The number of migrating and invading HN4 and Cal27 cells significantly decreased after ectopic expression of lncMX1–215 and increased in knockdown cells (Fig. 7a, b). Moreover, snail expression, a key transcription factor in epithelial-mesenchymal transition (EMT), was inhibited after overexpression of lncMX1–215, and vice versa (Fig. 7c). A pulmonary metastasis model was successfully established through tail vein injection, and lung metastases were diagnosed via pathological examination (Fig. 7d). The number of metastatic nodules in the lncMX1–215 overexpression group was notably decreased (Fig. 7e). Furthermore, ectopic expression of lncMX1–215 in SCC7 cells dramatically diminished lung metastasis in C3H mice (Additional file 3: Figure. S24b). To sum up, this study demonstrates that IFNα-induced lncMX1–215 expression can negatively regulate PD-L1 and galectin-9 expression by interrupting GCN5/H3K27ac binding in HNSCC (Fig. 7f).

## Discussion

Accumulating evidence indicates that lncRNAs participate in many physiological and pathological processes [22, 23]. In this study, we investigated lncRNA expression profiles in response to IFNα stimulation in HNSCC cells and identified a novel IFNα-induced lncRNA, lncMX1–215. Importantly, the lncMX1–215 level increased after IFNα treatment, and lncMX1–215 negatively regulated the expression of two key immunosuppression molecules, PD-L1 and galectin-9, which reversed IFNα-induced immunosuppression in HNSCC. Mechanistically, lncMX1–215 directly bound to GCN5, thereby interrupting H3K27 acetylation, resulting in inhibition of PD-L1 and galectin-9 transcription. Last, we demonstrated that lncMX1–215 acts as a tumor suppressor during tumor progression. This study elucidates a new regulation network in immunosuppression formation, which might provide novel insights into immune checkpoint blockade treatment in HNSCC.

Several cells and cytokines have been reported to be involved in immunosuppression, such as tumor-associated macrophages [24], cancer-associated fibroblasts [25], myeloid-derived suppressor cells [26], regulatory T cells, TGF-β [27], IL-6 [28], and interferon [9]. Clarifying the regulatory networks involved in immunosuppression formation in the tumor microenvironment is critical and will contribute to clinical treatment with ICBs. Among these factors, interferon plays a key role in immunosuppression because it can be produced by nearly all nucleated cells and its function impacts all cells in the microenvironment. Both our previous study and the ELISA results in this study confirmed secretion of IFNα and endogenous activation of interferon signaling. Interferon signaling is constitutively activated and promotes immune evasion of glioma cells [29]. Intriguingly, persistent IFN-I signaling can switch from immune stimulatory to immune suppressive activity, and blockade of sustained IFN-I signaling improves viral clearance during chronic infection [30]. IFNα drives the expression of other suppressive factors in addition to PD-L1, which might explain the failure of PD-1/PD-L1 blockade in 50% of melanoma patients [31]. Based on these studies, the mechanisms of IFNα-induced immunosuppression are pivotal in understanding immunosuppression regulation. Therefore, the present study focused on the molecular mechanism underlying immunosuppression in HNSCC, and the findings deepen understanding of immunosuppression development.

The complex, precise regulatory function of lncRNA in development and gene expression has greatly contributed to understanding of genome complexity and opened a new world for people to understand the complexity of life from the aspect of gene expression regulatory networks. In our study, lncMX1–215 was found to be a novel lncRNA involved in immunosuppression regulation. Lnc-lsm3b, an IFN-induced lncRNA, can compete with viral RNAs through binding of RIG-I monomers and contribute feedback that inactivates the innate RIG-I function at the late stage of the innate response [11]. Interestingly, a negative feedback loop to inhibit the interferon response was also established in our study. LncRNA-CD244 inhibits IFN-γ/TNF-α expression by repressing chromatin states in CD8 (+) T cells [32]. In our study, ten differentially expressed lncRNAs were found after lncRNA sequencing, but we failed to design specific primers for two of these lncRNAs; lncMX1–215 was identified according to its abundance, length, and fold change.

Mechanistic investigation of lncRNA depends heavily on cellular localization. Due to its localization in the nucleus, transcriptional regulation and chromatin modifications are the most promising interaction models for lncMX1–215. Treatment with Class I HDAC inhibitors resulted in rapid upregulation of histone acetylation of the PD-L1 gene, leading to enhanced and durable gene expression in melanoma [33]. Resveratrol and piceatannol promoted PD-L1 expression via the HDAC3/p300 axis in breast and colorectal cancer cells [34]. IFNα augmented the anti-tumor potential of HDAC inhibition in prostate cancer cells [35]. We found that IFNα and HDAC inhibitors had a synergistic effect on the expression of PD-L1 and galectin-9. This evidence suggests that histone acetylation plays a critical role in PD-L1 expression, but the type of acetylation still unknown. In our study, H3K27 acetylation was shown to alter the chromatin status and promote transcription of PD-L1 and galectin-9 in HNSCC. Therefore, we speculated that lncMX1–215 might exert its feedback function through histone modification. GCN5, also known as histone acetyltransferase 2A, is the primary enzyme catalyzing H3K27 acetylation [36]. Intriguingly, direct binding of lncMX1–215 to GCN5 was observed, which interrupted K3K27 acetylation. LncMX1–215 twined around GCN5 and impeded the acetylation enzyme activity. This might clearly explain the mechanism by which immunosuppression is mediated by IFNα-induced lncMX1–215 expression.

LncRNAs can be categorized as oncogenic or tumor suppressor lncRNAs according to their biological functions during carcinogenesis, such as the well-known MALAT1 [37], ANRIL [38] and H19 [39]. Since upregulation of lncMX1–215 can disrupt immunosuppression, which facilitates the killing of immune cells, overexpression of lncMX1–215 appears to be a protective factor in HNSCC. High lncMX1–215 levels inhibited the proliferation and metastasis capacity of HNSCC cells in vitro and in vivo. Moreover, lower lncMX1–215 expression was observed in HNSCC patients than in healthy controls, and low lncMX1–215 expression was correlated with a poor pathological grade. Furthermore, lncMX1–215 expression was negatively correlated with expression of immunosuppression molecules in paired HNSCC tissues. Based on all these results, lncMX1–215 can be considered a tumor suppressor in HNSCC.

## Conclusions

In this study, we identified a novel IFNα-induced lncRNA, lncMX1–215, whose expression was dependent on Stat1 phosphorylation. LncMX1–215 upregulation markedly inhibited PD-L1 and galectin-9 expression by interrupting GCN5-mediated H3K27 acetylation. The IFNα-lncMX1–215-GCN5-H3K27ac axis creates a novel feedback loop that disrupts immunosuppression formation in the tumor microenvironment. Our research provides new insight into the immunosuppression regulation network to enhance the antitumor effect of ICBs.

## Supplementary information

**Additional file 1: Table S1.** Primers and oligonucleotide sequences used for qPCR, siRNA, RACE, ChIP and RIP assays.

**Additional file 2: Table S2.** The correlation between H3K27ac and GCN5 expression and baseline characteristic in HNSCC patients.

**Additional file 3: Figure S1.** The IFNα concentration in medium supernatant from HNSCC cells, normal fibroblasts (NFs) and cancer-associated fibroblasts (CAFs) was measured via ELISA. The IFNα concentration was also detected after CDDP (2.5 μg/ml), 5-FU (10 μg/ml), cetuximab (200 ng/ml) and erlotinib (1.5 μM) treatment for 24 h in HNSCC cells. **Figure S2**. Differentially expressed lncRNAs were detected via sequencing after treatment with 200 ng/ml IFNα for 24 h. Differentially expressed lncRNAs were detected via sequencing after treatment with 200 ng/ml IFNα for 24 h. **Figure S3**. The nucleotide sequence of lncMX1-215 was identified using RACE (RACE for EGFR served as the positive control). **Figure S4**. LncMX1-215 chromatin location and encoding structure determined by RACE analysis was shown. **Figure S5**. LncMX1-215 localization was analyzed in Cal27 cells using PCR. U6 RNA and β-actin were used as the positive controls for nuclear RNA and cytoplasmic RNA, respectively. **Figure S6**. LncMX1-215 expression was detected in tumor and adjacent normal tissues from HNSCC patients. **Figure S7**. LncMX1-215 expression was analyzed after treatment with 200 ng/ml IFNα and 0.5 μM fludarabine for 24 h. **Figure S8**. LncMX1-215 expression was quantified using RT-PCR after stat1-specific siRNA transfection and then treatment with 200 ng/ml IFNα for 24 h. **Figure S9**. ChIP assays were performed using isotype IgG antibody after treatment with 200 ng/ml IFNα for 24 h. **Figure S10**. ChIP assays were conducted to analyze lncMX1-215 promoter binding under 200 ng/ml IFNα and 0.5 μM fludarabine treatment for 24 h. **Figure S11**. Cells were pretreated with 100 ng/ml rhGalectin-9 and then incubated with NK cells for 4 h. The specific lysis rate was measured using an LDH kit. **Figure S12**. **a** PD-L1 and acetylation of histone 3 were detected and quantified after treatment with 200 ng/ml IFNα or 15 μM SAHA for 24 h. **b** PD-L1, H3K27ac and H3K9ac were detected and quantified after the indicated SAHA treatment for 24 h. **c** PD-L1 and H3K27ac were detected and quantified after 15 μM SAHA treatment for the indicated time.# indicated the difference between combined group and each alone. *P < 0.05, and **P < 0.01. **Figure S13**. Galentin-9 expression was detected in HN4 and Cal27 cells after SAHA or MS-275 treatment for 24 h. **Figure S14** The promoter activity of PD-L1 and LGALS9 was measured after transfection with lncMX1-215 for 24 h and then 1.5 μM SAHA or 0.5 μM MS-275 treatment for 24 h in 293T cells.Fig. S15. After ectopic expression of GCN5 and vector or lncMX1-215 for 48 h in HN4 and Cal27 cells, ChIP assay was performed to analyze the binding to PD-L1 promoter using anti-GCN5 antibody. **Figure S16**. GCN5 and H3K27ac expression was detected using immunofluorescence in HN4 and Cal27 cells after lncMX1-215 transfection for 48 h. **Figure S17**. The expression of H3K27ac and GCN5 was detected using immunofluorescence in HNSCC TMA. **Figure S18**. RIP assays were performed with HN4 cells. **Figure S19**. A linear lncMX1-215 template was constructed after restriction enzyme digestion of the pcDNA3.1 recombinant vector. **Figure S20**. EdU assays were performed after transfection of HN4 and Cal27 cells with the indicated constructs; magnification: ×100. **Figure S21**. Tumors on the bilateral flank of nude mice were shown. **Figure S22**. TUNEL assays were conducted to assess the number of apoptotic cells in xenograft tumor sections; magnification: ×200. **Figure S23**. Ki-67 staining of xenograft tumor sections was performed. **Figure S24**. LncMX1-215 inhibited tumorigenesis and lung metastasis in SCC7-bearing mice. a SCC7-bearing xenografts were established in C3H mice and the tumors were resected and measured at experimental endpoint (n=5/group). b Lung metastasis assay was performed using SCC7 cells in C3H mice and the metastasis nodules were counted and analyzed (n=3/group).

## Data Availability

The dataset used and/or analyzed during the current study are available from the corresponding author on reasonable request.

## Abbreviations

ASO: Antisense oligonucleotide
ChIP: Chromatin Immunoprecipitation
CTLA-4: Cytotoxic T lymphocyte associated antigen
DAPI: 4′, 6-Diamidino-2-phenylindole
EdU: 5-Ethyny-2′-deoxyuridine assay
EMT: Epithelial-mesenchymal transition
FISH: Fluorescence in situ hybridization
H&E: Hematoxylin and Eeosin
HDAC: Histone deacetylase
HNSCC: Head and neck squamous cell carcinoma
ICBs: Immune checkpoint blockades
IHC: Immunohistochemistry
LAG-3: Lymphocyte-activation gene 3
LncRNA: Long non-coding RNA
NOK: Normal oral primary keratinocytes
PD-L1: Programmed cell death ligand 1
qRT-PCR: Quantitative real-time polymerase chain reaction
RACE: 5′- and 3′-Rapid amplification of cDNA ends
RIP: RNA Binding Protein immunoprecipitation
Stat1: Signal transducer and activator of transcription 1
TIGIT: T-cell immune receptor with immunoglobulin and ITIM domain
TIM-3: T cell immunoglobulin domain and mucin domain-3
TMA: Tissue microarrays
TUNEL: Terminal deoxynucleotidyl transferase dUTP nick end labelling
