# Supplementary material for: A novel IFNα-induced long noncoding RNA negatively regulates immunosuppression by interrupting H3K27 acetylation in head and neck squamous cell carcinoma
Source: Mol Cancer. 2020 Jan 6;19:4. doi: 10.1186/s12943-019-1123-y (PMC6943933; doi:10.1186/s12943-019-1123-y)
Supplement: Supplementary file 2 — Additional file 2: Table S2. The correlation between H3K27ac and GCN5 expression and baseline characteristic in HNSCC patients. [file 12943_2019_1123_MOESM2_ESM.docx]

Table S2. The correlation between H3K27ac and GCN5 expression and baseline characteristic in HNSCC patients

| Characteristics | Total patients N=70 (%) | H3K27ac expression | | | GCN5 expression | | |
| --- | --- | --- | --- | --- | --- | --- | --- |
|  |  | Low  N=42 | High  N=28 | *P value* | Low  N=56 | High  N=14 | *P value* |
| **Age** | | | | | | | |
| ＜60y | 32(45.7) | 22 | 10 | 0.170 | 24 | 8 | 0.337 |
| ≥60y | 38(54.3) | 20 | 18 |  | 32 | 6 |  |
| **Gender** | | | | | | | |
| Male | 42(60) | 25 | 17 | 0.921 | 31 | 11 | 0.113 |
| Female | 28(40) | 17 | 11 |  | 25 | 3 |  |
| **TNM stage** | | | | | | | |
| Ⅰ/Ⅱ | 31(44.3) | 23 | 8 | ***0.031*** | 29 | 2 | ***0.011*** |
| Ⅲ/Ⅳ | 39(55.7) | 19 | 20 |  | 27 | 12 |  |
| **Pathological grade** | | | | | | | |
| Well | 34(48.6) | 19 | 15 | 0.258 | 28 | 6 | 0.332 |
| Moderate | 25(35.7) | 18 | 7 |  | 21 | 4 |  |
| Poor | 11(15.7) | 5 | 6 |  | 7 | 4 |  |
