# Supplementary material for: A novel IFNα-induced long noncoding RNA negatively regulates immunosuppression by interrupting H3K27 acetylation in head and neck squamous cell carcinoma
Source: Mol Cancer. 2020 Jan 6;19:4. doi: 10.1186/s12943-019-1123-y (PMC6943933; doi:10.1186/s12943-019-1123-y)
Supplement: Supplementary file 3 — Additional file 3: Figure S1. The IFNα concentration in medium supernatant from HNSCC cells, normal fibroblasts (NFs) and cancer-associated fibroblasts (CAFs) was measured via ELISA. The IFNα concentration was also detected after CDDP (2.5 μg/ml), 5-FU (10 μg/ml), cetuximab (200 ng/ml) and erlotinib (1.5 μM) treatment for 24 h in HNSCC cells. Figure S2. Differentially expressed lncRNAs were detected via sequencing after treatment with 200 ng/ml IFNα for 24 h. Differentially expressed lncRNAs were detected via sequencing after treatment with 200 ng/ml IFNα for 24 h. Figure S3. The nucleotide sequence of lncMX1-215 was identified using RACE (RACE for EGFR served as the positive control). Figure S4. LncMX1-215 chromatin location and encoding structure determined by RACE analysis was shown. Figure S5. LncMX1-215 localization was analyzed in Cal27 cells using PCR. U6 RNA and β-actin were used as the positive controls for nuclear RNA and cytoplasmic RNA, respectively. Figure S6. LncMX1-215 expression was detected in tumor and adjacent normal tissues from HNSCC patients. Figure S7. LncMX1-215 expression was analyzed after treatment with 200 ng/ml IFNα and 0.5 μM fludarabine for 24 h. Figure S8. LncMX1-215 expression was quantified using RT-PCR after stat1-specific siRNA transfection and then treatment with 200 ng/ml IFNα for 24 h. Figure S9. ChIP assays were performed using isotype IgG antibody after treatment with 200 ng/ml IFNα for 24 h. Figure S10. ChIP assays were conducted to analyze lncMX1-215 promoter binding under 200 ng/ml IFNα and 0.5 μM fludarabine treatment for 24 h. Figure S11. Cells were pretreated with 100 ng/ml rhGalectin-9 and then incubated with NK cells for 4 h. The specific lysis rate was measured using an LDH kit. Figure S12. a PD-L1 and acetylation of histone 3 were detected and quantified after treatment with 200 ng/ml IFNα or 15 μM SAHA for 24 h. b PD-L1, H3K27ac and H3K9ac were detected and quantified after the indicated SAHA treatment for 24 h. c [file 12943_2019_1123_MOESM3_ESM.docx]

**Supplementary Figure legends**

**Fig. S1**. The IFNα concentration in medium supernatant from HNSCC cells, normal fibroblasts (NFs) and cancer-associated fibroblasts (CAFs) was measured via ELISA. The IFNα concentration was also detected after CDDP (2.5 μg/ml), 5-FU (10 μg/ml), cetuximab (200 ng/ml) and erlotinib (1.5 μM) treatment for 24 h in HNSCC cells.

**Fig. S2**. Differentially expressed lncRNAs were detected via sequencing after treatment with 200 ng/ml IFNα for 24 h.

**Fig. S3**. The nucleotide sequence of lncMX1-215 was identified using RACE (RACE for EGFR served as the positive control).

**Fig. S4**. LncMX1-215 chromatin location and encoding structure determined by RACE analysis was shown.

**Fig. S5**. LncMX1-215 localization was analyzed in Cal27 cells using PCR. U6 RNA and β-actin were used as the positive controls for nuclear RNA and cytoplasmic RNA, respectively.

**Fig. S6**. LncMX1-215 expression was detected in tumor and adjacent normal tissues from HNSCC patients.

**Fig. S7**. LncMX1-215 expression was analyzed after treatment with 200 ng/ml IFNα and 0.5 μM fludarabine for 24 h.

**Fig. S8.** LncMX1-215 expression was quantified using RT-PCR after stat1-specific siRNA transfection and then treatment with 200 ng/ml IFNα for 24 h.

**Fig. S9.** ChIP assays were performed using isotype IgG antibody after treatment with 200 ng/ml IFNα for 24 h.

**Fig. S10.** ChIP assays were conducted to analyze lncMX1-215 promoter binding under 200 ng/ml IFNα and 0.5 μM fludarabine treatment for 24 h

**Fig. S11.** Cells were pretreated with 100 ng/ml rhGalectin-9 and then incubated with NK cells for 4 h. The specific lysis rate was measured using an LDH kit.

**Fig. S12**. **a** PD-L1 and acetylation of histone 3 were detected and quantified after treatment with 200 ng/ml IFNα or 15 μM SAHA for 24 h. **b** PD-L1, H3K27ac and H3K9ac were detected and quantified after the indicated SAHA treatment for 24 h. **c** PD-L1 and H3K27ac were detected and quantified after 15 μM SAHA treatment for the indicated time.^#^ indicated the difference between combined group and each alone. * *P* < 0.05, and ** *P* < 0.01.

**Fig. S13.** Galentin-9 expression was detected in HN4 and Cal27 cells after SAHA or MS-275 treatment for 24 h.

**Fig. S14** The promoter activity of PD-L1 and LGALS9 was measured after transfection with lncMX1-215 for 24 h and then 1.5 μM SAHA or 0.5 μM MS-275 treatment for 24 h in 293T cells

**Fig. S15.** After ectopic expression of GCN5 and vector or lncMX1-215 for 48 h in HN4 and Cal27 cells, ChIP assay was performed to analyze the binding to PD-L1 promoter using anti-GCN5 antibody.

**Fig. S16.** GCN5 and H3K27ac expression was detected using immunofluorescence in HN4 and Cal27 cells after lncMX1-215 transfection for 48 h.

**Fig. S17.** The expression of H3K27ac and GCN5 was detected using immunofluorescence in HNSCC TMA.

**Fig. S18.** RIP assays were performed with HN4 cells.

**Fig. S19.** A linear lncMX1-215 template was constructed after restriction enzyme digestion of the pcDNA3.1 recombinant vector.

**Fig. S20.** EdU assays were performed after transfection of HN4 and Cal27 cells with the indicated constructs; magnification: ×100.

**Fig. S21.** Tumors on the bilateral flank of nude mice were shown.

**Fig. S22.** TUNEL assays were conducted to assess the number of apoptotic cells in xenograft tumor sections; magnification: ×200.

**Fig. S23.** Ki-67 staining of xenograft tumor sections was performed.

**Fig. S24.** LncMX1-215 inhibited tumorigenesis and lung metastasis in SCC7-bearing mice. **a** SCC7-bearing xenografts were established in C3H mice and the tumors were resected and measured at experimental endpoint (n=5/group). **b** Lung metastasis assay was performed using SCC7 cells in C3H mice and the metastasis nodules were counted and analyzed (n=3/group).

Fig. S1

|  |  |
| --- | --- |

Fig. S2

|  |  |  |  |
| --- | --- | --- | --- |
|  |  |  |  |

Fig. S3

| 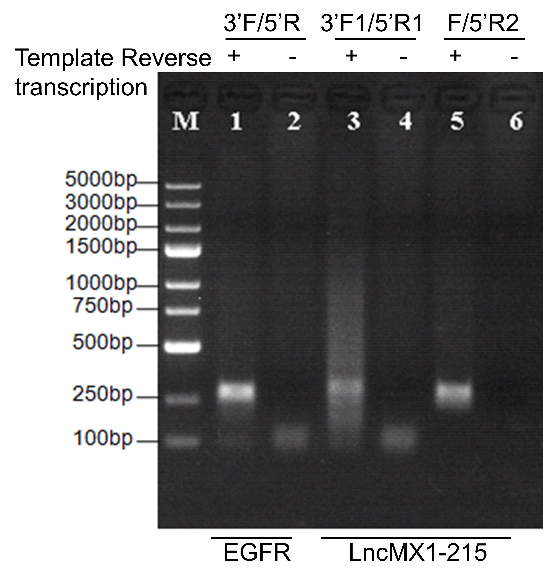 |
| --- |
| LncMX1-215 sequence (649 bp):  5’GCCATAAAATTTTGAGTAGAAAAATCCAGAAATTTGAAAATCAGTATCGTGGTAGAGAGCTGCCAGGCTTTGTGAATTACAGGACATTTGAGACAATCGTGAAACAGCAAATCAAGGCACTGGAAGAGCCGGCTGTGGATATGCTACACACCGTGACGGGTGAGTGCTCAGTTTCACCTCTGAGCATTGATTTCTAAAGAAAGGAAAGGTTCGAACCAAAGCCAGCACCAAACTTCAGCACTTTCCTCCTGGGGTGCATCCCACACCAACGAGCAAACCTCTCATTCTCCAGATGCCAAGTTGGTATTCAACAATTCAATTCAATTCTGACACTAACTACCCTCAGTCAGTGTGGACCCCATAGCTTAAGGGCTCAGTTCCACAACACTGGCCCCAACTACAAATGCCGGTCACAAGTCCCAGACCTCCTATTCTTCTGATGGACTGTTTATAAATCAAGGTTCTTGCGACCCATTCCTCAGGTCAACCAAGAACTCTGGAACACACTTCACTGACATTTACTGGTCTATTAGAAAGGATTTGATAAGGGTCACAAATGAAGCTGTTGGAGAGGCACATAGTAGAGGCCTGAACACAGAAGCTTCTGTCCCCACGGGGTTGGGGGCACCACCGTCATGGCACAGAGATG3’ |

Chr21:41445470-41449785

ENST00000486275/LncMX1-215

Chr21:41445470-41449785

ENST00000486275/LncMX1-215

Fig. S4

| 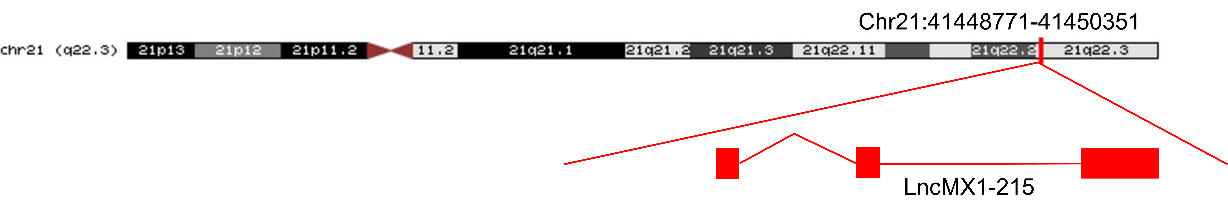 |
| --- |

Fig. S5

|  |
| --- |

Fig. S6

|  |
| --- |

Fig. S7

|  |
| --- |

Fig. S8

| 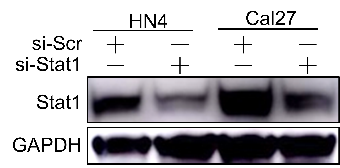 | 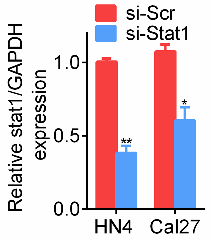 |  |
| --- | --- | --- |

Fig. S9

|  |  |
| --- | --- |

Fig. S10

|  |
| --- |

Fig. S11

| 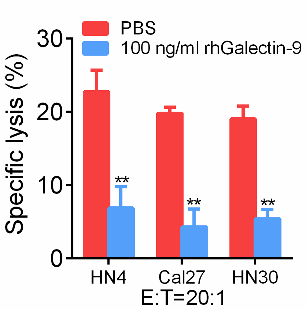 |
| --- |

Fig. S12

| 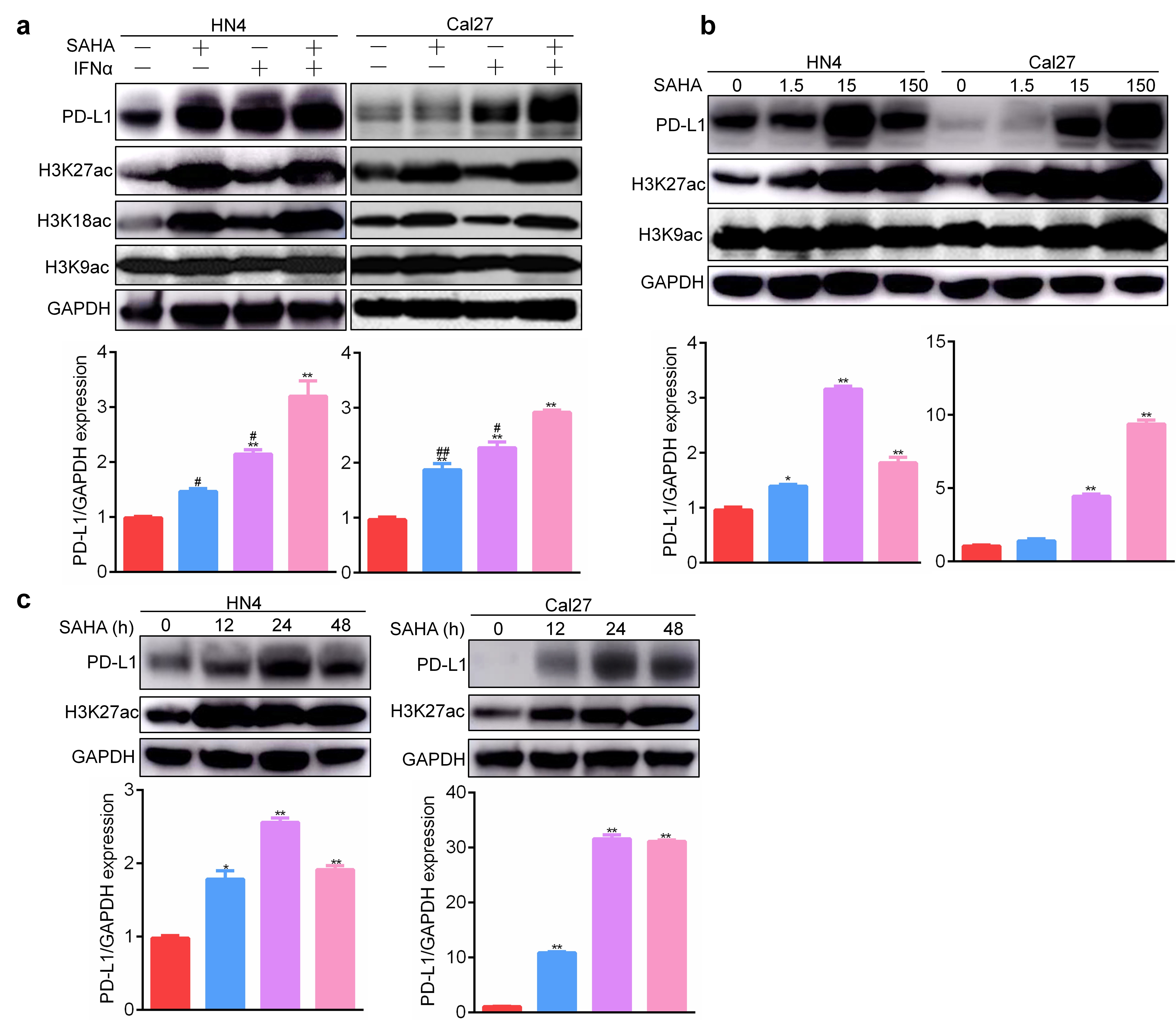 |
| --- |

Fig. S13

| 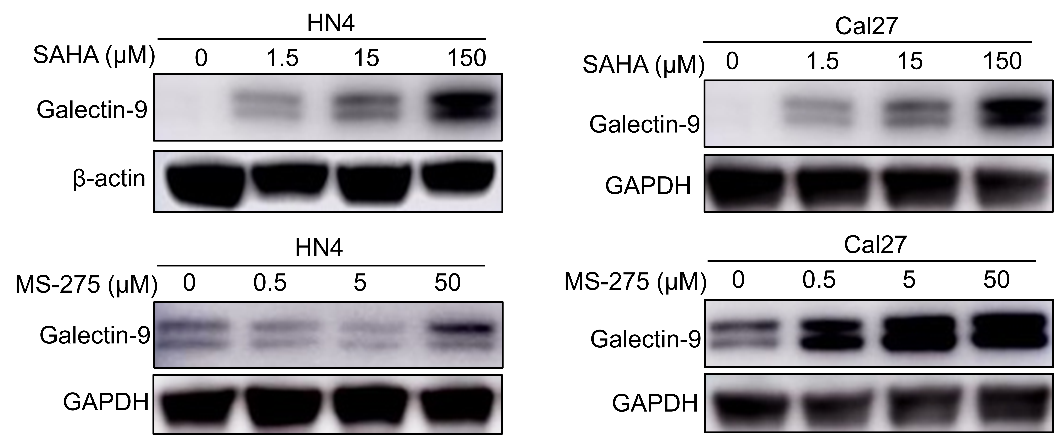  Fig. S14 | |
| --- | --- |
|  |  |

Fig. S15

|  |  |
| --- | --- |

Fig. S16

| 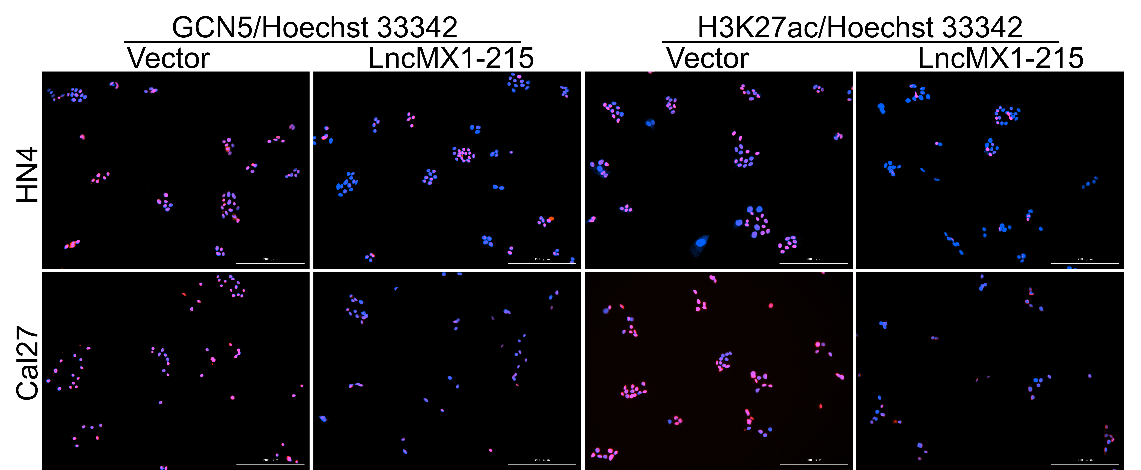 |
| --- |

Fig. S17

| 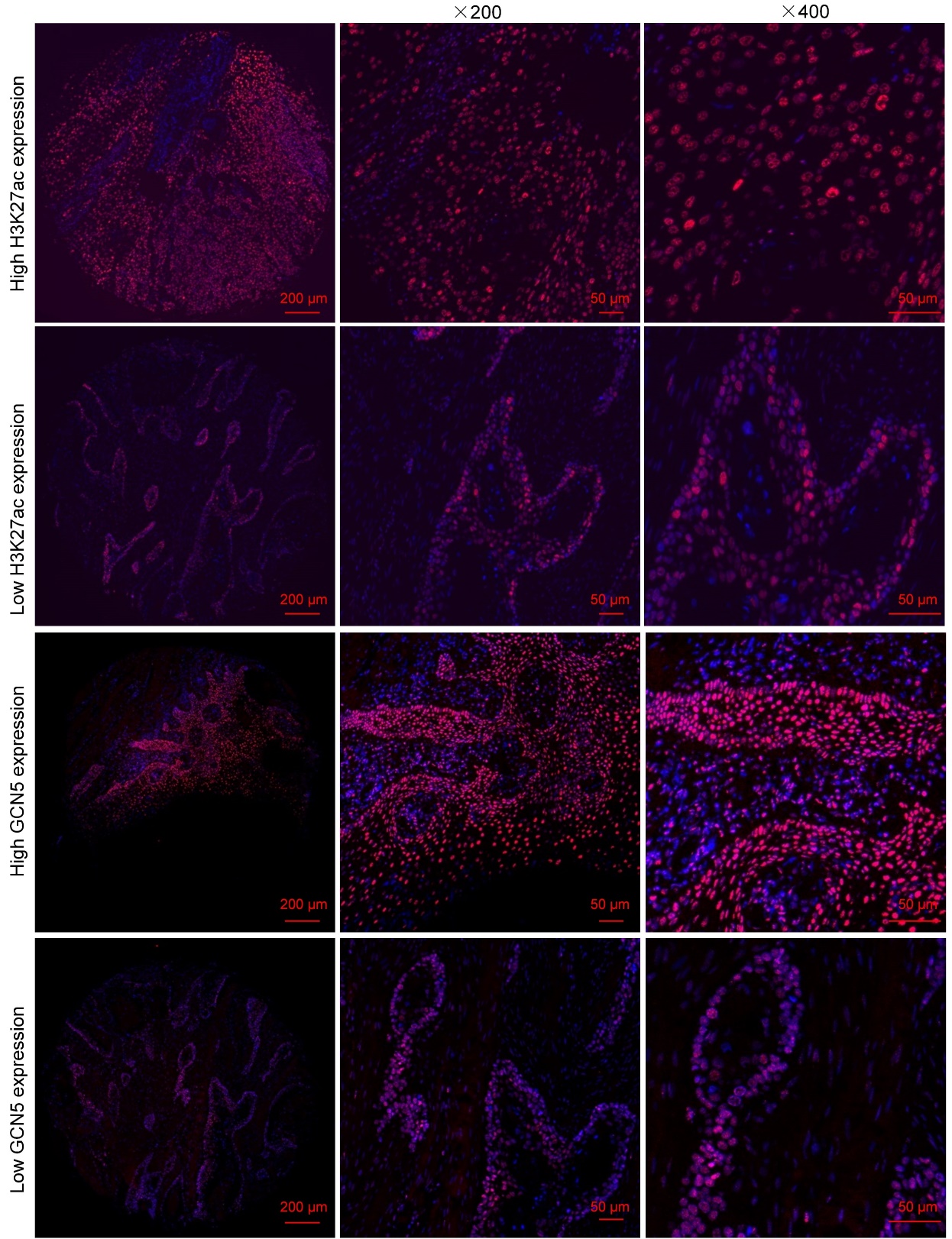 |
| --- |

Fig. S18

| 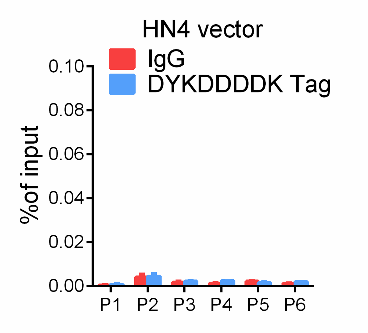 |
| --- |

Fig. S19

| 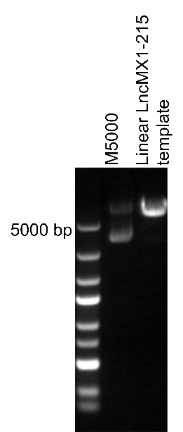 |
| --- |

Fig. S20

| 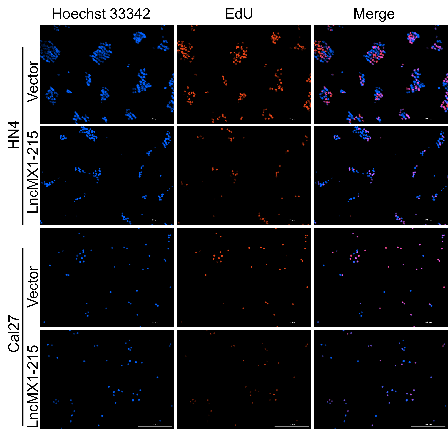 | 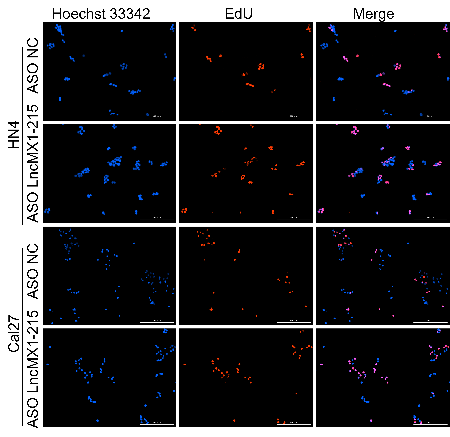 |
| --- | --- |

Fig. S21

| 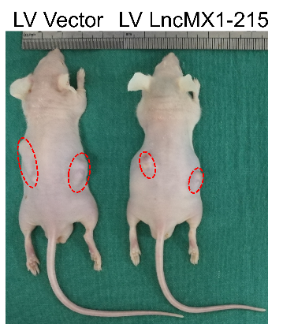 |
| --- |

Fig. S22

| 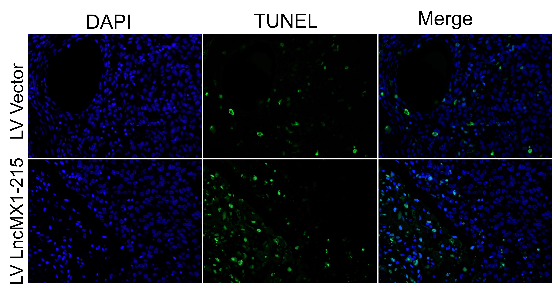 |
| --- |

Fig. S23

| 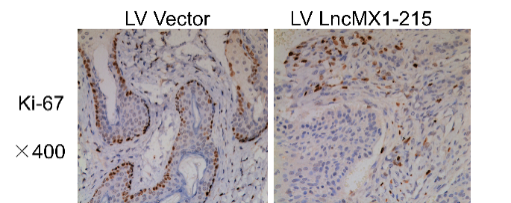 |
| --- |

Fig. S24

| 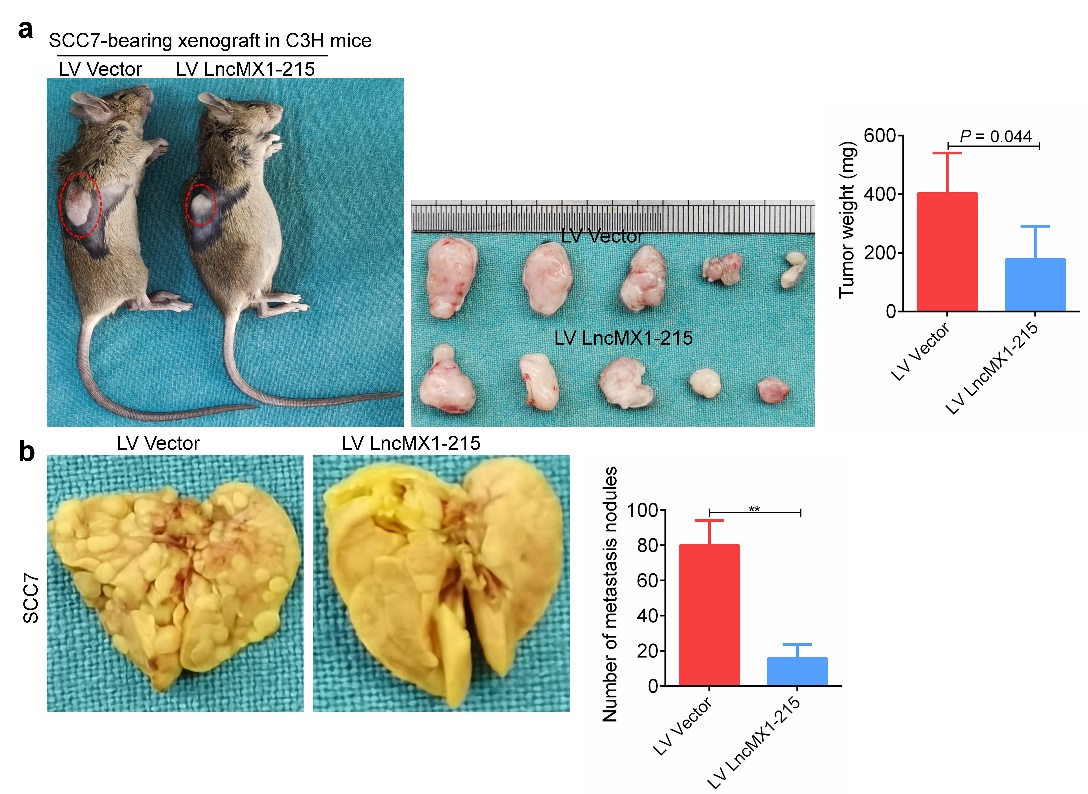 |
| --- |
